# High litter quality enhances plant energy channeling by soil macro‐detritivores and lowers their trophic position

**DOI:** 10.1002/ecy.70004

**Published:** 2025-02-23

**Authors:** Linlin Zhong, Thomas Larsen, Jing‐Zhong Lu, Stefan Scheu, Melanie M. Pollierer

**Affiliations:** ^1^ J.F. Blumenbach Institute of Zoology and Anthropology University of Göttingen Göttingen Germany; ^2^ Department of Archaeology Max Planck Institute of Geoanthropology Jena Germany; ^3^ Centre of Biodiversity and Sustainable Land Use University of Göttingen Göttingen Germany

**Keywords:** CSIA, earthworm ecological groups, energy channels, microbial community composition, resource quality, trophic position

## Abstract

Detritus‐based resources, that is, plant litter, are a major energy source for many living organisms and are considered to be a key determinant of primary production and nutrient cycling. Earthworms are among the most important macro‐detritivores in terrestrial food webs and play a crucial role in facilitating these processes in terrestrial ecosystems. Yet, the influence of litter quality on earthworm nutrition, and consequently on soil food web dynamics, has remained largely underexplored, mainly for methodological reasons. Here, we combined bulk and compound‐specific stable isotope analysis of amino acids to investigate the dietary contribution of different quality litter resources to earthworm species of different ecological groups. Our findings show that earthworms acquired most essential amino acids from bacterial (~60%) and plant (~30%) resources, with the latter increasing in importance with higher litter quality, resulting in lower trophic positions across earthworm species. The high bacterial contribution to earthworms corresponds to the dominance of bacteria in the experimental soil, suggesting that bacteria served as an important intermediate link in transferring detritus‐based resources to earthworms. Bacterial contributions were notably higher in the soil‐feeding earthworm species than in the litter‐feeding earthworm species, likely due to more pronounced ingestion of soil by soil‐feeding earthworms. Overall, our study indicates that a major group of soil macro‐detritivores, earthworms, receive detrital resources via the bacterial energy channel. Further, it underscores the important role of litter quality in shaping the trophic niches of detritivores, thereby influencing the overall structure of soil food webs.

## INTRODUCTION

Heterotrophic life on Earth crucially depends on energy and nutrients that are provided by primary production (Elton, 2001). The transfer of these resources to consumers is determined by feeding (trophic) interactions in food webs, and a multitude of ecosystem functions such as nutrient cycling, carbon sequestration, or decomposition depend on the trophic structure of food webs (Barnes et al., 2018). Across ecosystems, most of the primary production is not eaten by herbivores but instead fuels the food webs via detrital resources, that is, primarily plant leaf litter (Cebrian, 1999; Potapov et al., 2024). Detrital resources enter the food web through decomposition processes carried out by decomposer communities including fungi, bacteria, and litter‐feeding decomposer animals (Sterner & Elser, 2017). At the same time, the accessibility of detrital resources to consumers is likely influenced by the quality of the litter. This may be more pronounced in terrestrial than in aquatic systems due to the greater abundance of litter resources from vascular plants, higher accumulation of detritus, higher content of structural compounds such as lignin in litter, and more unevenly distributed resources in the former (Allan et al., 2021; Cebrian & Lartigue, 2004; Sterner & Elser, 2017; Tiegs et al., 2024). However, assessing trophic interactions in the detrital pathway in terrestrial systems is notoriously difficult due to the cryptic lifestyle of animals and the complexity of basal resources, restricting a more comprehensive understanding of fundamental ecosystem processes (Digel et al., 2014). This particularly applies to soil animals such as earthworms that feed on a mixture of dead plant material and soil, including microorganisms and microbial residues, making it hard to distinguish which fractions are actually assimilated (but see Larsen, Pollierer, et al., 2016).

As key macro‐detritivores in terrestrial ecosystems, earthworms occupy a central role in the soil food web (Zhou et al., 2022), altering soil structure, decomposition processes, and thereby nutrient availability to microorganisms and plants (Lavelle & Spain, 2002; Zhong et al., 2025). Due to their feeding on plant residues and associated microorganisms, earthworms couple plant, bacterial, and fungal energy channels, but relative proportions likely depend on resource quality. The recalcitrant compounds of low‐quality litter resources typically favor fungal abundance, whereas more accessible compounds in high‐quality litter resources tend to favor bacterial abundance (Rooney et al., 2006). Consequently, earthworms may incorporate low‐quality litter resources mainly via the fungal energy channel, whereas they likely incorporate high‐quality litter resources mainly via the bacterial and plant energy channels, with plant resources becoming more important with higher litter quality.

In addition, microbial communities are vertically structured in soil, with fungi typically dominating the organic or litter layer and bacteria dominating the mineral soil (Fierer et al., 2003; 2021 Lu & Scheu, 2021). Similarly, earthworms of different ecological groups also inhabit and feed on different soil layers. Therefore, due to the vertical distribution of earthworms and microorganisms, the energy channeling to earthworms presumably depends on their ecological group identity (Briones, 2014). The litter‐feeding epigeic and anecic earthworms likely assimilate litter resources via plant and fungal energy channels due to the higher fungal abundance in the litter layer (Lavelle & Spain, 2002). In contrast, the soil‐feeding endogeic species mostly feed in the upper mineral soil and thereby are more likely to incorporate litter‐derived resources via the bacterial energy channel due to the high abundance of bacteria in soil.

Due to their feeding strategies and burrowing activities, earthworms of different ecological groups may differentially affect the abundance and community composition of microorganisms (Hättenschwiler et al., 2005). For instance, increased microbial activity in earthworm casts, especially in those of epigeic and anecic earthworms, may increase the transformation of recalcitrant litter resources into more bioavailable molecules. This likely increases the transfer of detrital resources to higher trophic levels, in turn benefitting earthworm nutrition (“external rumen” hypothesis; Swift et al., 1979). In addition, anecic species likely also favor the growth of microorganisms by mixing litter and mineral soil (Edwards et al., 2022). However, there is limited empirical evidence on how different earthworm ecological groups respond to variations in litter quality and how their responses affect microbial community composition in soil.

Bulk stable isotope analysis is one of the widely used tools in soil food web analysis, allowing one to estimate trophic positions and the contribution of different resources to the nutrition of consumers (Potapov et al., 2019). The bulk isotope ratios of carbon (^13^C/^12^C) and nitrogen (^15^N/^14^N) in the consumer's body serve as biomarkers, with ^13^C/^12^C ratios informing about the use of carbon resources, for example, recently fixed versus microbially processed carbon, and ^15^N/^14^N ratios informing about trophic positions. However, accurate estimation of basal resources and trophic position can be difficult, in particular in soil food webs, as the basal resources are often mixed, for example, plant and microbial resources, making it difficult to determine the stable isotope composition of the consumers' food.

Compound‐specific isotope analysis (CSIA) of amino acids can overcome some of the limitations of bulk stable isotope analysis and provide more reliable insights into trophic niches of soil animals (Pollierer et al., 2019). In CSIA, the trophic position of organisms is estimated by the difference in δ^15^N values (as a measure of ^15^N/^14^N ratios) between “trophic” and “source” amino acids. Typically, the δ^15^N values of primary resources are represented in the δ^15^N values of “source” amino acids (e.g., phenylalanine [*Phe*]) while the trophic enrichment is reflected in the δ^15^N values of “trophic” amino acids (e.g., glutamine/glutamic acid [*Glu*]) (Chikaraishi et al., 2014). The δ^13^C values of amino acids offer a complementary perspective on the trophic niches. While bacteria, fungi, and plants synthesize essential amino acids (eAAs) via unique pathways that each exhibit distinct δ^13^C eAA fingerprints, metazoans lack the metabolic pathways to synthesize eAAs de novo. They therefore take up eAAs from these basal resources without or with minor modification, allowing one to estimate the relative contribution of basal resources to the diet of consumers (Larsen et al., 2009).

In this study, we investigated trophic niche differentiation among earthworm species of different ecological groups (epigeic, anecic, and endogeic) in response to litter of different quality, that is, litter materials forming a gradient of increasing lignin content and C‐to‐N ratio. The trophic niches of earthworms, that is, the use of basal resources and trophic position, were assessed by bulk stable isotope analysis and CSIA of amino acids. Hereafter, we use the term “energy channel” and “energy channeling” to describe the use of basal resources and to facilitate interpretation in light of soil food web theory (Moore & Hunt, 1988). We note that while eAAs serve as a proxy for energy flow, they do not directly equate to energy transfer. Additionally, we tested the effect of earthworm ecological groups and litter quality on the abundance of fungi and bacteria in litter and soil using phospholipid fatty acids (PLFA) analysis. We hypothesized that (1) earthworms feed more on plant‐derived resources and occupy a lower trophic position in high‐quality litter treatments, with this trend being stronger in epigeic and anecic than in endogeic earthworms. (2) Epigeic and anecic earthworms predominantly feed on plant and fungal resources due to the high abundance of fungi in litter, while endogeic earthworms mainly rely on bacterial resources due to the high abundance of bacteria in soil. (3) The fungal and bacterial abundance of litter and soil differs between litter treatments, with higher bacterial abundance in high‐quality litter and higher fungal abundance in low‐quality litter. (4) Earthworms modulate the abundance of fungi and bacteria in litter and soil, with this varying among earthworm ecological groups and with litter quality.

## MATERIALS AND METHODS

### Experimental setup

The microcosm experiment was set up in a full factorial design with two treatments: four litter types (wheat straw, horse manure, legume leaves [mixed leaves of *Trifolium pratense* and *Medicago sativa*], and rape leaves [*Brassica napus*]) and five earthworm species (*Eisenia fetida* [epigeic], *Lumbricus terrestris* [anecic], *Aporrectodea rosea* [endogeic], *Aporrectodea caliginosa* [endogeic], and *Allolobophora chlorotica* [endogeic], Appendix S1: Figure S1). Except *E. fetida*, all earthworm species used in this experiment were collected in March 2021 from a meadow close to the University of Göttingen, Germany (51°32′17.52″ N, 9°56′12.12″ E). The meadow was dominated by grasses (mainly *Lolium perenne* and *Arrhenatherum elatius*) and legumes (e.g., *Trifolium pratense*), but also herbs (e.g., *Taraxacum officinale*). We aimed to use common earthworm species of Central European grasslands belonging to three different ecological groups. As we only found endogeic and anecic but no epigeic earthworm species, we purchased *E. fetida* as a native European epigeic earthworm species occurring in rich organic soils (Obert & Vďačný, 2019). Its feeding strategy likely resembles that of other epigeic species associated with decaying plant materials such as rotting deadwood (e.g., *Dendrobaena octaedra*) or leaf litter (e.g., *Lumbricus rubellus*) (Edwards et al., 2022). Because *E. fetida* is commonly used in commercial vermiculture worldwide (Edwards et al., 2022), understanding its trophic ecology is of particular interest for agricultural management. *Eisenia fetida* was purchased from a culture shop (Wir haben Würmer, St. Gallen, Switzerland), where it was cultured on decomposing materials mixed with manure and plant residues. Juvenile and adult earthworms were collected. Adults were identified to species using Sims and Gerard (1999) and juveniles were ascribed to species based on pigmentation, arrangement of setae, and shape of prostomium.

The microcosms consisted of PVC tubes with an inner diameter of 10 cm and a height of 17 cm, covered with 200‐μm mesh at the bottom and surrounded by a transparent plastic sheet extending 10 cm above the top of the tube to prevent earthworms from escaping. The soil was taken from an agricultural field located in Relliehausen, Lower Saxony, Germany (51°46′42.4″ N, 9°41′42.7″ E), to a depth of 20 cm. The soil is characterized as Luvisol on loess with a loamy texture. The field was planted with wheat (C_3_ plant) when sampling and rotated with maize (C_4_ plant) and wheat in the years before. The soil was first sieved using a 4‐mm mesh to remove plant residues and then placed at −20°C for 10 days to kill existing earthworms. Each microcosm was filled with a mixture of 633 g of fresh weight sieved soil and 324 g of expanded clay (Bellandris Blähton, SAGAFLOR AG, Kassel, Germany) to improve soil structure. The soil moisture was kept at 70% of the maximum water holding capacity throughout the experiment. On top of the soil, 3 g dry litter was added initially corresponding to the litter treatments. Every 3 weeks, an additional 1 g of the respective litter was added to ensure continuous resource supply in the restricted space of the microcosms. Although earthworms are less restricted in their foraging range in the field and are therefore unlikely to deplete resources completely, we acknowledge that earthworms may face a more limited resource supply under natural conditions.

A total of 100 microcosms were established with the four litter types and five earthworm species (four litter treatments × five earthworm species × five replicates). Prior to the experiment, the litter was cut to the size of legume leaf litter to facilitate consumption by earthworms. Based on the litter C‐to‐N ratio, the quality of litter was ranked from high to low as follows: rape leaves, legume leaves, horse manure, and wheat straw (Appendix S1: Table S1). Five juvenile individuals of *E. fetida*, *L. terrestris*, *A. rosea*, *A. caliginosa*, and *A. chlorotica* were introduced into the respective treatments of the microcosms. The initial average total fresh biomass of *E. fetida*, *L. terrestris*, *A. rosea*, *A. caliginosa*, and *A. chlorotica* were 0.739 ± 0.003, 2.208 ± 0.009, 0.485 ± 0.004, 1.631 ± 0.005, and 0.858 ± 0.002 g, respectively. Microcosms were placed in darkness in a climate chamber at 20 ± 2°C and 70% humidity, watered four times a week based on gravimetric determination of the water loss, and randomized twice per week. At the end of the experiment, that is, after 18 weeks, microcosms were destructively sampled.

### Sampling

The soil was broken up by hand and the earthworms were picked, counted, and weighed. Then, earthworms were kept at −20°C for 1 day. Subsequently, the earthworms were squeezed under a stereomicroscope to empty their gut. Then, earthworms were washed and surface sterilized by placement in 70% ethanol for 10 min. Thereafter, earthworms were lyophilized and stored in a desiccator until further analysis. Litter materials were collected, lyophilized, weighed, and stored in a desiccator. The soil was sieved through 2‐mm mesh and stored at −20°C until further analyses.

### Bulk stable isotope analysis and CSIA of amino acids

Individual earthworms, dried litter material, and soil were subjected to both bulk stable isotope analysis and CSIA of amino acids with dual C and N stable isotope ratio analysis (Appendix S2: Section S1). The isotopic variation of C and N (δ*X*) was expressed as δ*X* (‰) = (*R*_sample_ − *R*_standard_)/*R*_standard_ × 1000, with *R* representing the ratio between the heavy and light isotopes (^13^C/^12^C or ^15^N/^14^N).

Amino acids were extracted as described by Larsen, Pollierer, et al. (2016; Appendix S2: Section S2) and then derivatized as described by Corr et al. (2007; Appendix S2: Section S2). Amino acid derivatives were then measured in triplicate using a gas chromatography combustion isotope ratio mass spectrometry system (GC‐C‐IRMS; Appendix S2: Section S3). We report all isotopic data in δ notation (‰). Stable isotope values of nitrogen and carbon in target amino acids were assessed independently. We obtained isotope values of 10 amino acids including alanine (*Ala*), asparagine/aspartic acid (*Asp*), glutamine/glutamic acid (*Glu*), glycine (*Gly*), isoleucine (*Ile*)*, leucine (*Leu*)*, methionine (*Met*)*, phenylalanine (*Phe*)*, threonine (*Thr*)* and valine (*Val*)* with the asterisks denoting eAAs.

### Phospholipid fatty acids analysis

PLFAs from soil and litter materials were extracted using a modified Bligh and Dyer method (Frostegård et al., 1993; Appendix S2: Section S4). PLFAs absolute abundances were calculated as nanomoles per gram dry weight of soil and litter. The PLFA 18:2ω6,9 was used as a fungal marker (Joergensen, 2022), while the saturated fatty acids i15:0, a15:0, i16:0, and i17:0 served as markers for Gram^+^ bacteria, and the fatty acids cy17:0, cy19:0, 16:1ω7, and 18:1ω7 as markers for Gram^−^ bacteria. Bacteria were represented by the sum of Gram^+^ and Gram^−^ bacteria. Total detected PLFAs (*n* = 28) were used to calculate PLFA absolute abundance.

### Statistical analysis

The variation in bulk δ^13^C and δ^15^N values, biomass of earthworms, as well as litter mass were analyzed using linear models, with earthworm species and litter treatments as explanatory categorical factors. Trophic position of earthworms as indicated by 𝛿^15^N values of amino acids (TP_CSIA_) was calculated using the following equation (Chikaraishi et al., 2014): TP_CSIA_ = [(δ^15^N_*Glu–*_ − δ^15^N_*Phe*_ − β)/TDF_*Glu‐Phe*_] + 1, with δ^15^N_*Glu*_ and δ^15^N_*Phe*_ representing the δ^15^N value of *Glu* and *Phe* from earthworms, respectively, β the difference between δ^15^N values in *Glu* and *Phe* of the primary producer (litter) in the food web, and TDF_*Glu*‐*Phe*_ (7.6 ± 1.2‰) the trophic discrimination factor at each shift of trophic level. We used specific β values from rape leaves (β = −7.5 ± 1.6‰) and legume leaves (β = −8.2 ± 1.4‰) for calculating the trophic position of earthworms. Due to the low δ^15^N concentration of amino acids in wheat straw, we could not obtain the δ^15^N value of *Glu* and *Phe*. Therefore, for earthworms sampled in the field and for those from the wheat straw and horse manure treatments, we used the β value for C_3_ plants (−8.4 ± 1.6‰; Chikaraishi et al., 2014). The variation in TP_CSIA_ of earthworms was analyzed using linear models, with earthworm species and litter treatments as explanatory variables. To evaluate the effect of litter quality on the TP_CSIA_ of earthworms, planned contrasts were designed to compare the differences in TP_CSIA_ of earthworms between treatments with wheat straw and each of the other litter treatments (Piovia‐Scott et al., 2019). To predict the biosynthetic origin of eAAs in earthworms, we used the fingerprinting approach as described in Larsen et al. (2009). Briefly, we applied linear discriminant analysis (LDA) with δ^13^C values of the eAAs including *Ile*, *Leu*, *Phe*, *Thr*, and *Val*. We excluded *Met* and *Lys* because the chromatography of these amino acids was not satisfactory in all samples. We used eAAs δ^13^C values of bacteria, fungi, and plants obtained from Larsen et al. (2009), Larsen, Pollierer, et al. (2016), and Pollierer et al. (2019), as well as those of rape and legume leaves from this experiment as classifier variables to identify the contribution of the basal resources to the diet of earthworms in the LDA. A leave‐one‐out cross‐validation approach was used to ensure the basal resource groups (plant, fungi, and bacteria) were statistically different. Statistical differences were confirmed by a high classification accuracy (98.9%, Appendix S1: Table S2, Figure S2). We then ran multivariate analyses of variance (MANOVAs) for the LDA classification to inspect the effects of earthworm species and litter treatments on the use of basal resources by earthworms. To estimate the proportion of basal resources used by earthworms, we ran Bayesian mixing models based on eAAs δ^13^C values centered on the mean value of all eAAs. Because the dynamic range of mean‐centered δ^13^C values of *Ile* and *Thr* was too small to be informative, we excluded these eAAs and only used the three most informative eAAs (*Leu*, *Phe*, and *Val*). The Bayesian mixing models, which explicitly included earthworm species and litter treatments as fixed nested factors, were set to run for 300,000 iterations (burn‐in = 200,000) on three parallel Monte Carlo Markov chains with a thinning interval of 100 using non‐informative priors. The models were evaluated using the Gelman–Rubin diagnostic (*R̂* values <1.05).

The fungal‐to‐bacterial ratio and abundance of fungi and bacteria in soil and litter were analyzed separately using linear models with earthworm species and litter treatments as explanatory variables. Only the mole percentages of PLFA higher than 0.2% of total PLFAs were included in the analyses.

All statistical analyses were run in R version 4.3.0 (R Core Team, 2023). Bayesian mixing models were run using the “MixSIAR” package (Stock et al., 2018); LDAs were conducted using the “MASS” package (Ripley et al., 2024). Statistical results of linear models and MANOVAs were obtained using the “vegan” (Oksanen et al., 2024) and “stats” packages (R Core Team, 2023). Planned contrasts were performed using the “emmeans” package (Lenth, 2024). Data were transformed (log [earthworm biomass and litter mass] or mean‐centered [δ^13^C values of the eAAs]) prior to analyses when necessary to improve normality and homogeneity of variance. All figures were drawn using “ggplot2.”

## RESULTS

### Changes in litter mass and earthworm biomass

The final litter mass varied with litter treatments and depended on earthworm species (Table 1, Appendix S1: Figure S3a). Across litter treatments, litter mass significantly decreased during the experiment, with the decrease being more pronounced in litter of higher quality (−72.7 ± 2.9%, −68.6 ± 3.0%, and −10.0 ± 0.5% for rape leaves, legume leaves, and wheat straw, respectively). The reduction in litter mass was particularly strong in treatments with the epigeic species *E. fetida* and the anecic species *L. terrestris*, with the latter also strongly reducing the mass of horse manure (−82.6 ± 2.7%). The endogeic species *A. caliginosa* and *A. chlorotica* to a similar extent increased the mass loss of rape and legume leaves, on average by 62.3 ± 1.1%.

**TABLE 1:** *F*‐ and *p*‐values of analyses of variance on the effect of litter treatments (wheat straw, horse manure, legume leaves, rape leaves), earthworm species (*Eisenia fetida*, *Lumbricus terrestris*, *Aporrectodea rosea*, *Aporrectodea caliginosa*, *Allolobophora chlorotica*) and their interaction (E × L) on the remaining litter mass, the difference between initial and final earthworm biomass, bulk δ^15^N and δ^13^C values of earthworms, as well as the trophic position and use of basal resources of earthworms.

Response	Factor	df	*F‐value*	*P‐value*
Final litter mass	Litter treatment (L)	3	1511.93	**<0.001**
	Earthworm species (E)	4	288.81	**<0.001**
	E × L	12	46.28	**<0.001**
	Residuals	80		
Change in biomass	Litter treatment (L)	3	105.07	**<0.001**
	Earthworm species (E)	4	179.83	**<0.001**
	E × L	12	40.07	**<0.001**
	Residuals	80		
δ^15^N	Litter treatment (L)	3	16.15	**<0.001**
	Earthworm species (E)	4	351.64	**<0.001**
	E × L	12	6.31	**<0.001**
	Residuals	80		
δ^13^C	Litter treatment (L)	3	40.39	**<0.001**
	Earthworm species (E)	4	88.02	**<0.001**
	E × L	12	6.886	**<0.001**
	Residuals	80		
Trophic position	Litter treatment (L)	3	28.33	**<0.001**
	Earthworm species (E)	4	210.42	**<0.001**
	E × L	12	2.944	**0.002**
	Residuals	80		
Basal resources	Litter treatment (L)	3	17.88	**<0.001**
	Earthworm species (E)	4	29.32	**<0.001**
	E × L	12	5.418	**<0.001**
	Residuals	80		

*Note*: Significant effects are given in bold (*p* < 0.05).

Changes in earthworm biomass also varied significantly with litter treatments (Table 1, Appendix S1: Figure S3b). The biomass of *E. fetida* increased by 78.0 ± 5.2% and 91.5 ± 4.4% in treatments with legume and rape leaves, respectively. Similarly, the biomass of *L. terrestris* increased in the treatments with legume leaves, rape leaves, and horse manure by 171.8 ± 11.7%, 135.5 ± 13.1%, and 63.2 ± 8.1%, respectively. Further, the biomass of *A. caliginosa* increased in each of the litter treatments, with the highest increase in the treatment with legume leaves (106.7 ± 10.6%). By contrast, the biomass of *A. chlorotica* only increased in the treatment with legume leaves (52.6 ± 4.4%), while the biomass of *A. rosea* remained constant across all litter treatments.

Juveniles and cocoons were produced by *E. fetida* in all litter treatments, with the number of offspring (both juveniles and cocoons) being over 20 times higher in the legume and rape leaves treatments than in the horse manure and wheat straw treatments (Appendix S1: Table S3). Similarly, the reproductive behavior of endogeic and anecic earthworm species was observed in the legume leaf treatment, with a similar number of offspring in species of both groups (endogeic: new juveniles: 0.6 ± 0.4, cocoon: 3.0 ± 0.8; anecic: new juvenile: 1.0 ± 0). Surprisingly, neither juveniles nor cocoons were produced by endogeic or anecic earthworm species in the rape leaf treatment. Conversely, *A. rosea* produced similar numbers of juveniles and cocoons in the horse manure and wheat straw treatments.

### Bulk stable isotope composition of earthworms, litter, and soil

Litter δ^13^C values were highest in wheat straw (−28.7 ± <0.1‰), followed by horse manure (−29.2 ± <0.1‰), legume leaves (−29.9 ± <0.1‰), and rape leaves (−31.4 ± <0.1‰; Appendix S1: Figure S4). By contrast, the δ^15^N values of litter were highest in rape leaves (4.5 ± 0.1‰), intermediate in horse manure (3.1 ± 0.1‰), and lowest in legume leaves and wheat straw (−0.5 ± 0.1‰ for both; Appendix S1: Figure S4). Soil δ^13^C and δ^15^N values were constant across litter treatments, averaging −27.1 ± <0.1‰ and 5.2 ± 0.1‰, respectively.

In general, δ^13^C values of the epigeic earthworm species *E. fetida* and the anecic species *L. terrestris* were similar to those of soil, whereas in the endogeic earthworm species *A. rosea*, *A. caliginosa*, and *A. chlorotica*, they were higher than those of soil, on average, being increased on average by 2.2 ± 0.1‰ (Appendix S1: Figure S5b). All earthworm species were enriched in ^13^C compared with litter (Appendix S1: Figure S5a), but this was more pronounced in the endogeic (+4.8 ± 0.1‰) than in epigeic (+3.6 ± 0.1‰) and anecic species (+2.8 ± 0.2‰). Further, δ^13^C values of earthworms also varied with litter treatments. Compared with the wheat straw treatment, the δ^13^C values of *E. fetida* were generally lower in the other litter treatments, and this was most pronounced in the rape leaves treatment (−3.3 ± 0.3‰; Appendix S1: Figure S5b). Similarly, compared with the wheat straw treatment, the δ^13^C values of *L. terrestris* were lower in the rape leaves treatment (−1.1 ± 0.3‰). By contrast, δ^13^C values of the endogeic earthworm species did not differ among litter treatments.

Similar to δ^13^C values, the δ^15^N values of earthworms varied between earthworm species, but this depended on litter treatments (Table 1, Appendix S1: Figure S4). Compared with the wheat straw treatment, δ^15^N values of *E. fetida* were lower in each of the other litter treatments, with the effect being most pronounced in the legume leaves treatment (−3.5 ± 0.5‰). By contrast, δ^15^N values of *L. terrestris* were 2.6 ± 0.5‰ higher in rape leaves than in the wheat straw treatment. Earthworm δ^15^N values of endogeic species were little affected by litter treatments, except in *A. caliginosa*; in this species, δ^15^N values were slightly lower in the horse manure than in the rape leaves treatment. Each of the earthworm species was enriched in ^15^N compared with litter (Appendix S1: Figure S5a); δ^15^N values of *E. fetida* exceeded those in litter by 10.1 ± 0.6‰, while in the anecic and endogeic species they were on average only 3.3 ± 0.2‰ higher than those in litter. In addition, δ^15^N values of *E. fetida* were higher than those in soil, while in the anecic and endogeic earthworm species, they were similar to those in soil, rape leaves, and horse manure (Appendix S1: Figure S5b).

### Trophic positions of earthworms derived from CSIA of amino acids

**FIGURE 1:**
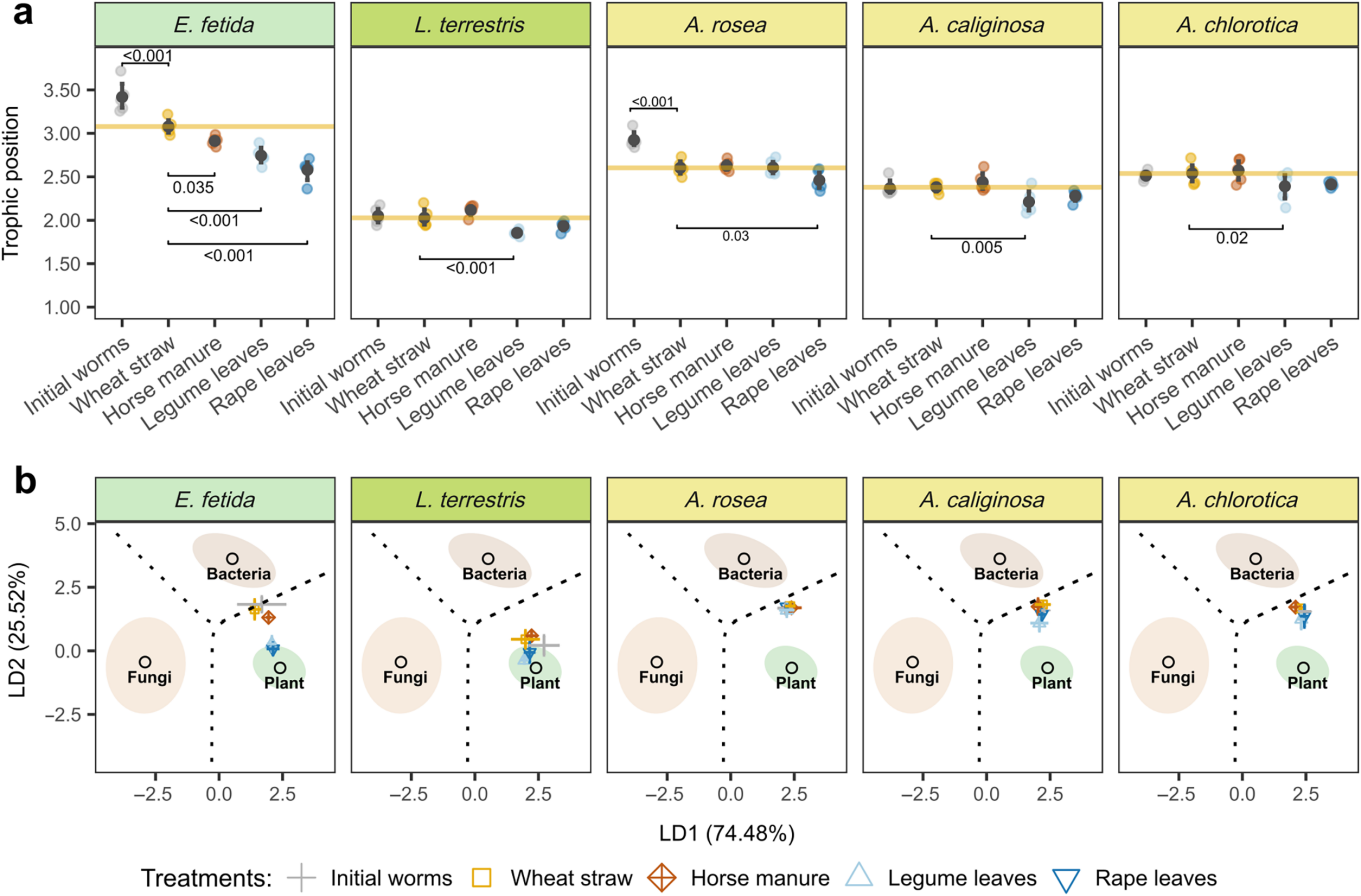
(a) Trophic position (TP_CSIA_; arithmetic means ± 95% CIs) of five earthworm species in different litter treatments (wheat straw, horse manure, legume leaves, and rape leaves) and before placement into the microcosms as calculated from δ^15^N values of glutamic acid and phenylalanine. TP_CSIA_ of 1, 2, and 3 represents plants, primary, and secondary decomposers, respectively. The yellow line indicates the mean TP_CSIA_ in the wheat straw treatment; dots with representative color indicate individual specimens. The number in the panel indicates significant differences to the wheat straw treatment based on planned contrasts between litter types and wheat straw nested within each earthworm species (*p* < 0.05; for detail see Appendix S1: Table S4). (b) Linear discriminant analysis (LDA) of δ^13^C values in essential amino acids of the five earthworm species (arithmetic means ± 95% CIs) in the treatment with wheat straw (yellow; square), horse manure (brown; diamond), legume leaves (light blue; triangle) and rape leaves (dark blue; angle down) and before placement into the microcosms (initial; gray, cross). Training data for plants, bacteria, and fungi were used as endmembers to predict the biosynthetic origin of essential amino acids in earthworms. Ellipses represent 95% CIs of each resource group, and dashed lines represent the decision boundaries.

Trophic positions of earthworms as indicated by TP_CSIA_ varied in an interactive way with litter treatments and earthworm species (Table 1, Figure 1a). Prior to the experiment, the averages of TP_CSIA_ of *E. fetida*, *L. terrestris*, *A. rosea*, *A. caliginosa*, and *A. chlorotica* were 3.4 ± 0.1, 2.0 ± <0.1, 2.9 ± <0.1, 2.4 ± <0.1 and 2.5 ± <0.1, respectively (Figure 1a). Compared with wheat straw treatments, the TP_CSIA_ of all earthworm species except *A. rosea* was lower in the presence of legume leaves, with the decline being most pronounced in *E. fetida*, in which the TP_CSIA_ decreased by 0.3 ± 0.1 compared with the wheat straw treatment. Similarly, in the presence of rape leaves, the TP_CSIA_ was also lower in all earthworm species compared with the wheat straw treatment, but the decline was only significant in *E. fetida* and *A. rosea*, in which the TP_CSIA_ decreased by 0.5 ± 0.1 and 0.1 ± 0.1, respectively. Notably, the presence of horse manure only decreased the TP_CSIA_ of *E. fetida* by 0.2 ± 0.1 compared with the wheat straw treatment.

### Basal resources of earthworms inferred from amino acid ^13^C fingerprinting

**FIGURE 2:**
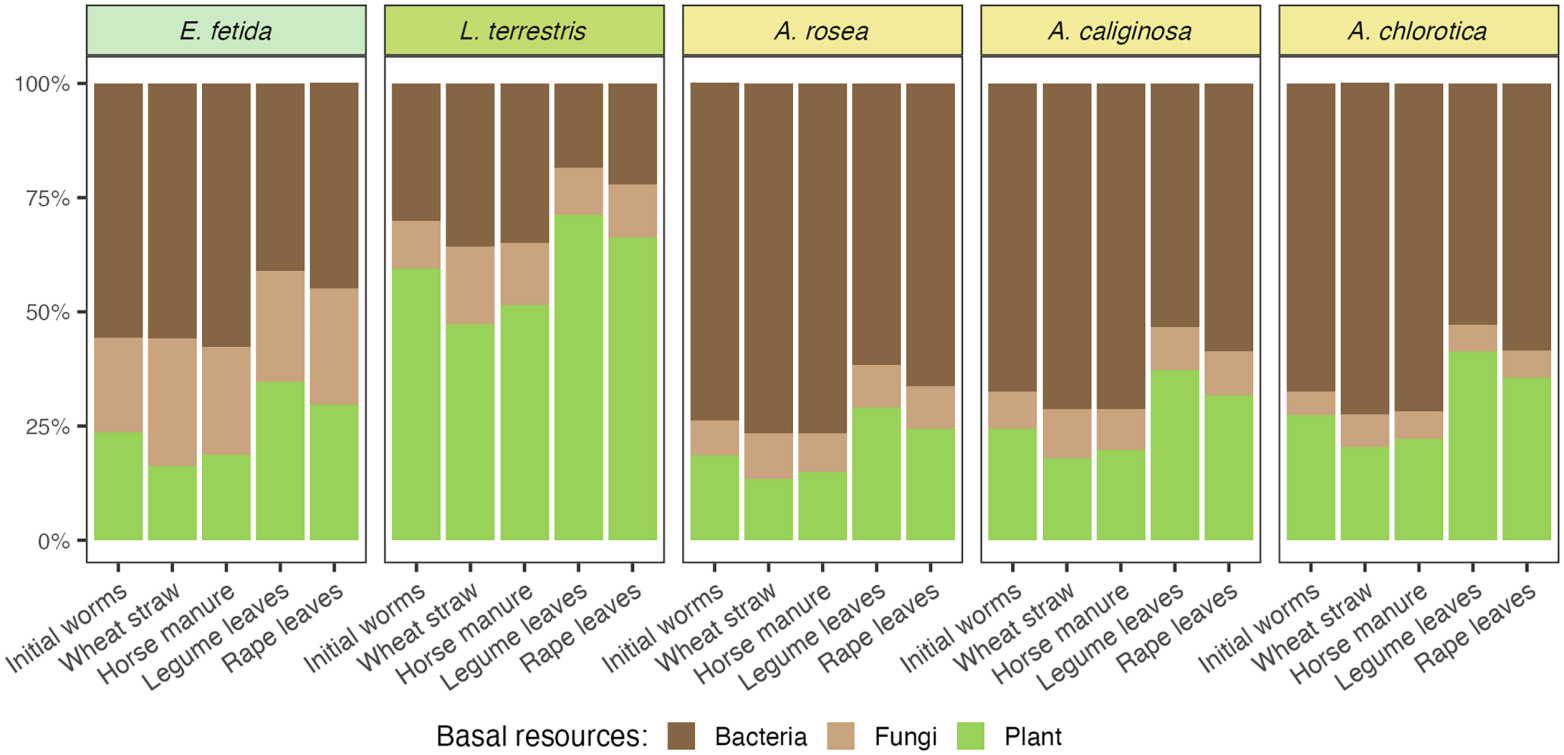
Relative contribution of bacterial (dark brown), fungal‐ (light brown) and plant‐derived essential amino acids (light green) to the diet of earthworms in the treatments with wheat straw, horse manure, legume leaves, and rape leaves as well as of earthworms before placement into the microcosms (initial) as estimated by Bayesian mixing models based on mean‐centered δ^13^C values of the three most informative essential amino acids: leucine, phenylalanine, and valine.

The use of basal resources by earthworms significantly differed between earthworm species (MANOVA, Table 1, Figure 1b). The epigeic earthworm species *E. fetida* and the anecic earthworm species *L. terrestris* mainly relied on plant‐derived resources, whereas the endogeic earthworm species *A. rosea*, *A. caliginosa*, and *A. chlorotica* relied more on bacterial‐derived resources. However, the use of basal resources by earthworm species also depended on litter treatments (Table 1). In the presence of legume and rape leaves, the epigeic species *E. fetida* and the endogeic species *A. caliginosa* and *A. chlorotica* shifted toward the use of plant‐derived resources, while they shifted to more bacterial‐derived resources in the presence of wheat straw and horse manure. The litter treatment effect on the use of basal resources was most pronounced in *E. fetida* as indicated by the fingerprinting approach (Figure 1b). The mixing models further indicated that all earthworm species studied consumed more plant‐derived resources in the legume and rape leaves treatments compared with the wheat straw and horse manure treatments (Figure 2, Appendix S1: Figure S6). In the wheat straw and horse manure treatments, *E. fetida* mainly relied on bacterial (51.0 ± 3.4%) and less on plant‐derived resources (24.6 ± 3.4%), while it shifted to plant‐derived resources in the treatments with legumes and rape leaves (32.3 ± 2.5%). In line with the fingerprinting results, *L. terrestris* predominantly relied on plant‐derived resources (59.1 ± 4.4%), with this being most pronounced in the treatments with legume and rape leaves. The endogeic earthworm species mainly relied on bacterial resources (66.7 ± 2.0%), but slightly shifted toward plant resources in the treatments with legume and rape leaves. Additionally, the mixing model showed that the relative contribution of fungal‐derived resources to epigeic (24.4 ± 1.2%) and anecic (12.6 ± 1.2%) species was higher than in endogeic species (on average 8.0 ± 0.1%).

### Microbial community structure in soil and litter

**FIGURE 3:**
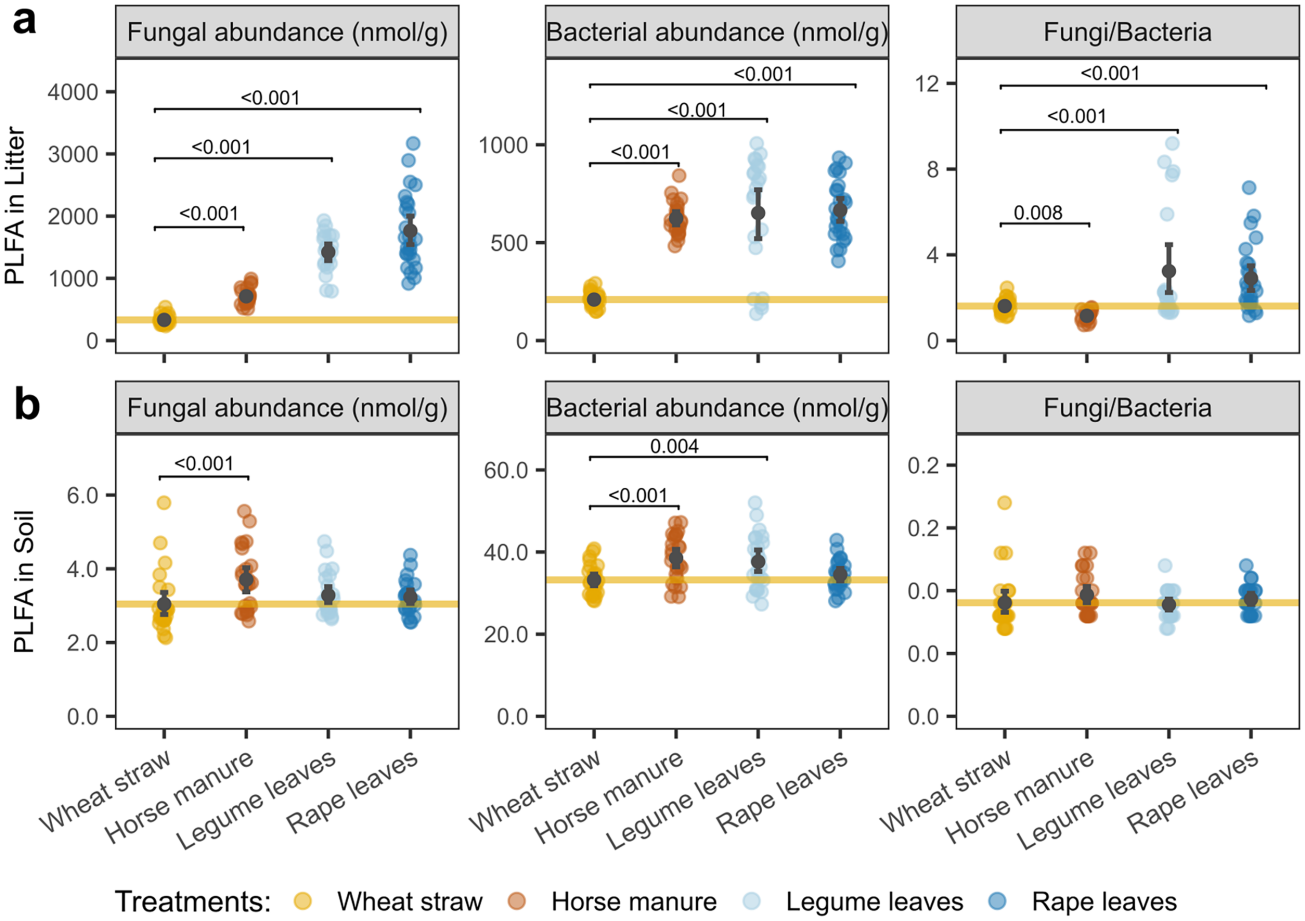
(a) Absolute abundance of fungal and bacterial fatty acid markers, and the fungi‐to‐bacteria ratio in (a) litter and (b) soil. The yellow line indicates the mean value in the wheat straw treatment. Dots with representative color indicate individual specimens, and the black dots indicate the mean values; the error bar indicates the 95% CIs. The number in the panel indicates significant differences to the wheat straw treatment based on planned contrast between each other litter type and wheat straw (*p* < 0.05; for detail see Appendix S1: Table S6). PLFA, phospholipid fatty acids.

The fungal‐to‐bacterial ratio was highest in legume and rape leaves, intermediate in wheat straw, and lowest in horse manure. However, the fungal and bacterial abundance in litter varied with litter type and depended on earthworm species (Appendix S1: Table S5). The absolute abundance of fungi in litter generally increased with litter quality, with the fungal abundance in rape leaves being highest and in wheat straw being lowest (Figure 3a). The bacterial abundance in legume and rape leaves as well as in horse manure was consistently higher than that in wheat straw. In addition, the fungal abundance in litter also depended on earthworm species, in particular in rape leaves, where the presence of *E. fetida* and *L. terrestris* increased the fungal abundance by 55.2% compared with endogeic species (Appendix S1: Figure S7a). By contrast, the presence of *L. terrestris* decreased the bacterial abundance in legume leaves by 77.3% compared with the other earthworm species.

In contrast to litter, the fungal‐to‐bacterial ratio in soil was similar across litter treatments, with bacterial markers being generally more abundant than fungal markers (Figure 3b). However, the abundance of both bacteria and fungi in soil varied in an interactive way with litter type and earthworm species (Appendix S1: Table S5). The fungal abundance was higher in the treatment with horse manure than that with wheat straw, especially in the presence of *E. fetida* and *L. terrestris*, where the fungal abundance increased by an average of 39.3% (Figure 3b, Appendix S1: Figure S7b). Additionally, the presence of *E. fetida* and *L. terrestris* generally resulted in higher soil bacterial abundance than that of endogeic earthworm species, in particular in the legume leaf and horse manure treatments.

## DISCUSSION

The majority of energy and nutrients originating from primary production is processed via decomposition of detritus (Cebrian, 1999). Earthworms are among the most prominent decomposer animals in terrestrial systems; however, their trophic niche, that is, the proportions of plant, fungal, and bacterial resources they utilize, is masked by their ingestion of high amounts of mixed resources such as soil and leaf litter. Here, we applied stable isotope analyses to uncover the trophic niches of earthworms belonging to different ecological groups and to elucidate how they respond to different litter quality. All earthworm species incorporated litter resources via microbial energy channels, as indicated by both bulk‐tissue and eAAs ^13^C values. eAAs of earthworms predominantly originated from bacteria (~60%), whereas fungi contributed little (~10%), corresponding to the dominance of bacteria in the experimental soil. Further, higher litter quality strengthened the plant energy channel and resulted in lower trophic positions of earthworms, indicating a crucial role of resource quality in shaping the trophic niches of soil animals.

### Litter quality as a driving factor of earthworm growth and bulk stable isotope composition

Increased litter mass loss in the presence of high‐quality litter suggests that high‐quality litter decomposed more quickly and was consumed more intensively by earthworms. This is supported by higher earthworm biomass gain in the treatments with rape and legume leaves than in those with wheat straw and horse manure. The more balanced stoichiometry between high‐quality litter resources (C‐to‐N ratio of ~13) and earthworms (C‐to‐N ratio of ~4), compared with low‐quality litter (C‐to‐N ratio of ~93), likely enables earthworms to assimilate plant litter resources more efficiently (Sterner & Elser, 2017), resulting in higher biomass gain. Notably, compared with treatments with endogeic earthworm species, the litter mass loss was more pronounced in the presence of the epigeic *E. fetida* and the anecic *L. terrestris*, suggesting a more intense consumption of high‐quality litter by the latter. This was corroborated by higher biomass gains of *L. terrestris* and a higher number of offspring in *E. fetida* (Appendix S2: Table S3) in the high‐quality litter treatments. Intensive consumption of high‐quality litter by *L. terrestris* is further supported by bulk ^15^N values, which were about 3‰ enriched compared with litter, suggesting predominant assimilation of litter resources. By contrast, the high ^15^N values of *E. fetida* likely stemmed from its pre‐experimental diet of high ^15^N values. Thus, bulk ^15^N values of *E. fetida* should be interpreted cautiously.

All earthworm species were enriched in ^13^C by 2‰–7‰ compared with litter, which exceeds the 0.5‰–1‰ enrichment per trophic level in non‐detrital systems. This phenomenon, coined “detrital shift” (Potapov et al., 2019), has been attributed to the consumption of microbially processed organic matter or the uptake of ^13^C‐enriched leaf litter compounds (Pollierer et al., 2009). The shift was more pronounced in the endogeic (>4‰) than in the epigeic and anecic species (2‰–4‰), suggesting that the epigeic and anecic species relied more on litter resources, whereas the endogeic species depended more on microbial‐derived resources. Soil ^13^C values typically increase with increasing soil depth due to the accumulation of old carbon resources heavily processed by microorganisms (Ehleringer et al., 2000). Therefore, the stronger enrichment in ^13^C in endogeic earthworms likely reflects their higher reliance on old carbon resources compared with epigeic and anecic species (Ferlian et al., 2014). This is supported by the overlapping ^15^N values of endogeic earthworms and organic matter in soil and is consistent with earlier findings that endogeic species assimilate more old carbon than epigeic and anecic species (Briones et al., 2005). Notably, in treatments with wheat straw and horse manure, *E. fetida* had higher ^13^C values than in treatments with legume and rape leaves, indicating a greater reliance on ^13^C‐enriched resources such as microbially processed carbon in low‐quality litter treatments.

### Effects of litter quality on trophic niches of earthworms

Supporting our first hypothesis, earthworms in the high‐quality litter treatments generally occupied lower trophic positions and relied more on plant‐derived resources, suggesting selective feeding on litter resources. This was most pronounced in the epigeic species, as indicated by the reduction in its trophic position. However, the strong shift in trophic position in *E. fetida* may be partially attributed to its pre‐experimental diet as noted above. Notably, the high trophic position of *L. terrestris*, *A. caliginosa*, and *A. chlorotica* in low‐quality litter treatments resembled their initial trophic position. This could be due to either little incorporation of new resources in low‐quality litter treatments or to the incorporation of similar resources in the soil they were sampled from and in the low‐quality treatments. The fact that the biomass of these three earthworm species in the horse manure treatment, and of *A. caliginosa* also in the wheat straw treatment, significantly increased during the experiment argues against little turnover/incorporation of tissue carbon, but instead suggests the incorporation of similar resources in the experiment and the soil they were sampled from. By contrast, the biomass of *L. terrestris* and *A. chlorotica* in the wheat straw treatment did not increase during the experiment, suggesting little tissue turnover, thus necessitating careful interpretation of their stable isotope values.

Nevertheless, all studied earthworm species shifted to higher proportions of plant resources in the rape and legume leaves treatments compared with treatments with wheat straw and horse manure. This likely reflects increased consumption and assimilation of plant resources in the high‐quality litter treatments, as has also been shown in aquatic animals such as pond snails and crustaceans (Zhang et al., 2018). By contrast, wheat straw and horse manure were rich in lignin and holocellulose that restrict the use of plant resources by detritivores. The digestion of these recalcitrant litter compounds requires cellulases and extracellular hydrolytic and oxidative enzymes, which earthworms are unlikely to synthesize themselves. Instead, they depend on microorganisms to produce these enzymes, as suggested by the “external rumen” hypothesis (Swift et al., 1979). Additionally, gut microbes may also supplement the nutrition of earthworms under nutrient‐deprived conditions (Larsen, Ventura, et al., 2016). However, the short retention time (2–24 h) of food resources during the earthworm gut passage likely does not allow a crucial role of gut microbes in aiding the digestion of litter materials (Drake & Horn, 2007; Zeibich et al., 2019); thereby, the “external rumen” hypothesis is more likely. This is supported by higher trophic positions of earthworms in wheat straw and horse manure treatments, indicating that the assimilation of microbial resources drives the elevation of the trophic position of decomposer animals (i.e., trophic inflation) (Steffan et al., 2017). More intermediate microbial trophic steps in response to lower diet quality could be a universal pattern, as similar results have been reported for a number of soil animals such as isopods and ants as well as for aquatic animals (Helms et al., 2021; van der Lee et al., 2021; van Straalen, 2023).

The trophic niches of the earthworms in our study aligned with their assigned ecological groups. Both the TP_CSIA_ results and the observed shifts toward greater utilization of plant resources in high‐quality litter treatments among epigeic and anecic species confirmed that these species predominantly consume litter‐derived resources (Edwards et al., 2022). The higher consumption of litter resources was accompanied by a higher proportion of fungal eAAs in the epigeic and anecic earthworms due to the high fungal abundance in litter. Although the TP_CSIA_ of endogeic species was generally higher than that of epigeic and anecic species, it also varied among endogeic species, indicating trophic niche differentiation (Capowiez et al., 2024; Zhong, Larsen, et al., 2024). For instance, high‐quality litter significantly lowered the TP_CSIA_ of *A. caliginosa* and *A. chlorotica* but not that of *A. rosea*, indicating a more pronounced shift toward the use of plant‐derived resources in the former, as also evidenced by the increase in their biomass in the high‐quality litter treatments. Despite these intragroup differences, trophic niche variation was less pronounced within endogeic species than across earthworm ecological groups, implying that the ecological grouping of earthworms is a robust indicator of trophic niche separation among earthworms.

### Basal resource use and energy channeling

The fungal‐to‐bacterial ratio was higher in litter than in soil, and this corroborated with the fungal energy channel being more important in the epigeic and anecic than in endogeic species, partially supporting our second hypothesis. However, our findings suggest that fungi only moderately contribute to earthworm nutrition. Rather, litter‐derived resources were predominantly incorporated via the plant (~30%) and bacterial (~60%) energy channels, with lower quality litter resulting in a higher contribution of the bacterial energy channel. As the bacterial channel is perceived as “fast energy channel” (Coleman et al., 1983), the higher reliance on the bacterial channel likely leads to faster transfer and higher loss of energy along food chains in the low diet quality scenario. This is consistent with high nutrient leaching and fast mineralization of soil organic matter in intensively managed agricultural systems lacking input of high‐quality residues such as mulch material (Corbeels et al., 2006; de Vries et al., 2006).

The predominance of bacterial eAAs in earthworms corresponded to the dominance of bacteria in our experimental soil. Although the epigeic earthworm species *E. fetida* is thought to predominantly feed and assimilate plant resources (Lavelle & Spain, 2002), it also contained a significant amount of bacterial eAAs in its tissue, which, as noted above, may have originated from the pre‐experimental diet. However, the anecic and endogeic earthworms also contained high proportions of eAAs from bacteria, indicating that bacteria or their residues in soil serve as important food resources. This was particularly evident in *L. terrestris*, which pulls litter into its burrows and feeds on microbially colonized litter within the soil matrix (Lavelle & Spain, 2002). As bacteria are often associated with small soil particles such as clay, earthworms ingest bacteria and bacterial residues when feeding on soil (Hemkemeyer et al., 2018). The latter form an important component of soil organic matter and, by breaking up these soil aggregates during the gut passage, earthworms may be able to access these resources (Angst et al., 2022). By contrast, it is unlikely that earthworms effectively digest living bacteria, since bacterial biomass and the number of bacterial cells in soil do not decrease during the earthworm gut passage (Scheu, 1987; Schönholzer et al., 1999). Based on pulse ^13^C labelling of plants and CSIA of amino acids, a previous study documented that earthworms, in particular endogeic species, were only little enriched in ^13^C from recent plant photosynthates, but substantial amounts of bacterial‐derived eAAs were found in their tissue (Zhong et al., 2024). This also suggests that earthworms rely on bacterial residues associated with older soil organic matter rather than on living bacterial biomass. Given the dominance of the fungal energy channel in soil food webs (Pausch et al., 2016), the predominant incorporation of plant and bacterial resources uniquely positions earthworms in soil food webs and may explain why earthworms reach high biomass and dominate energy processing in many soil food webs (Zhou et al., 2022). The influence of litter quality on dietary preferences and, in turn, on the channeling of energy and nutrients may be highly relevant for agricultural systems and management decisions, as this may feedback to ecosystem functions and services.

### Effects of litter quality and earthworm species on the abundance of fungi and bacteria

In part supporting our third hypothesis, the abundance of fungi and bacteria in litter and soil varied with litter treatments. In litter, total microbial abundance and fungal‐to‐bacterial ratio were higher in the rape and legume leaves than in the horse manure and wheat straw treatments, likely relating to lower C‐to‐N ratio and lignin content of rape and legume leaves. In soil, the abundance of fungi and bacteria generally varied little with litter quality, likely reflecting that leachates from litter of different quality are similar (Hensgens et al., 2021). However, the abundance of fungi and bacteria was higher in the soil of the horse manure than in the wheat straw treatments due to the higher microbial abundance in the former, presumably reflecting the more intensive incorporation of manure into the soil by earthworms.

Providing evidence for our fourth hypothesis, earthworms differentially modulated the abundance of fungi and bacteria in litter and soil. In litter, the abundance of fungi in rape leaves was higher in the presence of the epigeic *E. fetida* and the anecic *L. terrestris* than in the presence of endogeic species. Presumably, the litter‐feeding epigeic and anecic species increased the availability of nitrogen by casting and excreting mucus and urine in litter, thereby facilitating the exploitation of litter resources by fungi. The increased fungal abundance may have contributed to the increased contribution of fungi to the diet of epigeic and anecic earthworms, as indicated by CSIA, suggesting a positive feedback loop.

Variations in the abundance of fungi and bacteria with earthworm species were stronger in soil than in litter, presumably reflecting the translocation of litter resources into the soil by earthworms (bioturbation). In particular, *E. fetida* and *L. terrestris* increased the abundance of bacteria (and microorganisms in total), and this was likely due to the incorporation of litter materials by these species into the soil, with this being more pronounced in horse manure, legume leaves, and rape leaves treatments. Similar to *L. terrestris*, *A. caliginosa*, as endogeic species, also increased the fungal abundance in soil in the horse manure treatment. In fact, *A. caliginosa* is known to feed on the dung of vertebrate herbivores, reflecting that its grouping as endogeic species is simplistic (Barley, 1959; Capowiez et al., 2024).

## CONCLUSIONS

To quantify the effects of detrital quality on the trophic position and energy channeling of earthworms as major decomposer animals, we quantified the contributions of plant, bacterial, and fungal basal resources to their nutrition in response to a gradient of litter quality. Earthworms incorporated litter resources mainly via bacterial (~60%) and plant (~30%) energy channels, with soil‐feeding species being more strongly linked to the bacterial energy channel than litter‐feeding species, presumably due to the predominance of bacteria in soil. Interestingly, within the earthworm species, shifts in the abundance of fungi and bacteria in the litter little affected energy channeling into earthworms. Rather, high litter quality increased the assimilation of plant litter by earthworms, at least at the given time scale, resulting in lower trophic positions and supporting the view that bottom‐up forces structure decomposer communities. By contrast, lower quality litter resources increased the contribution of microorganisms to the nutrition of earthworms, potentially reflecting a general pattern of microorganisms as trophic intermediates in response to low diet quality. Overall, our study points to the fundamental role of plant resource quality as an indicator of energy channeling and trophic position in animals, which is likely a universal pattern in detrital food webs.

## CONFLICT OF INTEREST STATEMENT

The authors declare no conflicts of interest.

## Supporting information


Appendix S1.

Appendix S2.

## Data Availability

Data (Zhong, 2024) are available in Figshare at https://doi.org/10.6084/m9.figshare.26520172.v2.
