# Supplementary material for: High litter quality enhances plant energy channeling by soil macro‐detritivores and lowers their trophic position
Source: Ecology. 2025 Feb 23;106(2):e70004. doi: 10.1002/ecy.70004 (PMC11848239; doi:10.1002/ecy.70004)
Supplement: Supplementary file 1 — Appendix S1. [file ECY-106-e70004-s002.pdf]

Journal: Ecology

Manuscript type: Article

High litter quality enhances plant energy channelling by soil macro-detritivores and lowers their trophic position

Authors: Linlin Zhong*, Thomas Larsen, Jing-Zhong Lu, Stefan Scheu, Melanie M. Pollierer

*Corresponding author (E-mail: linlin.zhong@gwdg.de)

Appendix S1

Table S1 Lignin, holocellulose, α-cellulose, carbon and nitrogen concentrations, and C-to-N ratio and bulk stable isotope values ($\delta^{13}C$ and $\delta^{15}N$) of wheat straw, horse manure, legume leaves and rape leaves used in the experiment; n, number of replicates.

Litter type	Variable	mean ± se	n
Wheat straw	**Lignin (%)**	20.76 ± 1.79	5
Horse manure		36.53 ± 1.13	3
Legume leaves		16.22 ± 0.48	5
Rape leaves		5.13 ± 0.67	5
Wheat straw	**Holocellulose (%)**	71.79 ± 0.55	5
Horse manure		65.1 ± 2.3	3
Legume leaves		34.32 ± 0.85	5
Rape leaves		24.92 ± 1.12	5
Wheat straw	**α-cellulose (%)**	38.55 ± 1.38	5
Horse manure		33.49 ± 0.45	3
Legume leaves		20.75 ± 1.22	5
Rape leaves		15.12 ± 1.28	5
Wheat straw	**C (%)**	45.09 ± 2.5	5
Horse manure		43.68 ± 2.81	3
Legume leaves		42.73 ± 0.87	5
Rape leaves		43.25 ± 0.37	5
Wheat straw	**N (%)**	0.34 ± 0.02	5
Horse manure		1.00 ± 0.04	3
Legume leaves		2.82 ± 0.06	5
Rape leaves		3.52 ± 0.05	5
Wheat straw	**C/N**	134.42 ± 0.77	5
Horse manure		43.52 ± 0.69	3
Legume leaves		15.15 ± 0.03	5
Rape leaves		12.28 ± 0.06	5
Wheat straw	**$\delta^{13}C_{bulk}$**	-28.67 ± 0.01	5
Horse manure		-29.24 ± 0.05	3
Legume leaves		-29.9 ± 0.11	5
Rape leaves		-31.37 ± 0.02	5
Wheat straw	**$\delta^{15}N_{bulk}$**	-0.43 ± 0.42	5
Horse manure		3.06 ± 0.09	3
Legume leaves		-0.66 ± 0.11	5
Rape leaves		4.46 ± 0.03	5

Table S2 Reclassification table of basal resource groups (bacteria, fungi and plants) generated using the leave-one-out cross-validation method based on linear discriminant analysis (LDA). Correct reclassifications are shown in bold, and the accuracy of samples correctly reclassified for each basal resource group is shown in the right column.

		Initial groups		
		Bacteria	Fungi	Plants
Reclassification	Bacteria	**12**	0	0
	Fungi	0	**36**	0
	Plants	0	1	**40**
	accuracy (%)	100	97.3	100

Table S3 Number of juvenile earthworms and cocoons produced by earthworm species (*Eisenia fetida*, *Lumbricus terrestris*, *Aporrectodea rosea*, *Aporrectodea caliginosa*, *Allolobophora chlorotica)* in different litter treatments (wheat straw, horse manure, legume leaves and rape leaves).

Litter types	Earthworms	Number of produced juveniles	Number of produced cocoons
Legume leaves	*A. caliginosa*	0	3.0 ± 0
Legume leaves	*A. chlorotica*	0.5 ± 0.5	3.8 ± 1.4
Legume leaves	*A. rosea*	1.0 ± 1.0	1.3 ± 0.7
Legume leaves	*E. fetida*	50.0 ± 27.8	17.0 ± 8.5
Legume leaves	*L. terrestris*	0	1.0 ± 0
Rape leaves	*A. caliginosa*	0	0
Rape leaves	*A. chlorotica*	0	0
Rape leaves	*A. rosea*	0	0
Rape leaves	*E. fetida*	111.0 ± 23.2	23.4 ± 6.3
Rape leaves	*L. terrestris*	0	0
Horse manure	*A. caliginosa*	0	0
Horse manure	*A. chlorotica*	0	1.0 ± 0
Horse manure	*A. rosea*	5.0 ± 0	1.0 ± 0
Horse manure	*E. fetida*	1.5 ± 0.5	0
Horse manure	*L. terrestris*	0	0
Wheat straw	*A. caliginosa*	0	0
Wheat straw	*A. chlorotica*	0	0
Wheat straw	*A. rosea*	6.0 ± 0	1.0 ± 0
Wheat straw	*E. fetida*	4.5 ± 3.5	2.0 ± 2.0
Wheat straw	*L. terrestris*	0	0

Table S4 Estimated marginal means based on linear models for effects of litter treatments (wheat straw, horse manure, legume leaves and rape leaves) and earthworm species (*Eisenia fetida*, *Lumbricus terrestris*, *Aporrectodea rosea*, *Aporrectodea caliginosa*, *Allolobophora chlorotica)* on earthworm trophic position; df, degrees of freedom; CL, 95% confidence level estimated based on the "emmeans" package; P-values are derived from contrast against wheat straw nested within each earthworm species.

Litter types	Earthworms	Means	Standard error	df	lower.CL	upper.CL	P-values (contrast vs. wheat straw)
Horse manure	*E. fetida*	-0.055	0.026	100	-0.106	-0.004	**0.035**
Legume leaves	*E. fetida*	-0.115	0.026	100	-0.166	-0.064	**<0.001**
Rape leaves	*E. fetida*	-0.177	0.026	100	-0.228	-0.126	**<0.001**
Initial worms	*E. fetida*	0.104	0.026	100	0.053	0.155	**0.000**
Horse manure	*L. terrestris*	0.044	0.026	100	-0.007	0.095	0.091
Legume leaves	*L. terrestris*	-0.088	0.026	100	-0.139	-0.037	**0.001**
Rape leaves	*L. terrestris*	-0.047	0.026	100	-0.098	0.004	0.071
Initial worms	*L. terrestris*	0.011	0.026	100	-0.040	0.062	0.676
Horse manure	*A. rosea*	0.009	0.026	100	-0.042	0.060	0.719
Legume leaves	*A. rosea*	0.001	0.026	100	-0.050	0.052	0.967
Rape leaves	*A. rosea*	-0.057	0.026	100	-0.108	-0.006	**0.028**
Initial worms	*A. rosea*	0.116	0.026	100	0.065	0.167	**<0.001**
Horse manure	*A. caliginosa*	0.024	0.026	100	-0.027	0.075	0.358
Legume leaves	*A. caliginosa*	-0.075	0.026	100	-0.126	-0.024	**0.005**
Rape leaves	*A. caliginosa*	-0.044	0.026	100	-0.095	0.007	0.092
Initial worms	*A. caliginosa*	-0.005	0.026	100	-0.056	0.046	0.837
Horse manure	*A. chlorotica*	0.013	0.026	100	-0.038	0.064	0.607
Legume leaves	*A. chlorotica*	-0.061	0.026	100	-0.112	-0.010	0.020
Rape leaves	*A. chlorotica*	-0.050	0.026	100	-0.101	0.001	0.057
Initial worms	*A. chlorotica*	-0.009	0.026	100	-0.060	0.042	0.724

Table S5 F- and P-values of analyses of variance on the effect of litter treatments (wheat straw, horse manure, legume leaves, rape leaves), earthworm species (*Eisenia fetida*, *Lumbricus terrestris*, *Aporrectodea rosea*, *Aporrectodea caliginosa*, *Allolobophora chlorotica*) and their interaction (E x L) on the absolute abundance of fungi and bacteria in soil and litter as measured by phospholipid fatty acid analysis. Significant effects are given in bold (P < 0.05); df, degrees of freedom.

Response	Factor	df	F-value	P-value
**Fungal abundance in soil**	Litter treatment (L)	*3*	5.39	**0.002**
	Earthworm species (E)	*4*	4.03	**0.005**
	E x L	*12*	2.83	**0.003**
	Residuals	*77*		
**Bacterial abundance in soil**	Litter treatment (L)	*3*	15.65	**<0.001**
	Earthworm species (E)	*4*	28.85	**<0.001**
	E x L	*12*	3.56	**<0.001**
	Residuals	*77*		
**Fungal abundance in litter**	Litter treatment (L)	*3*	130.28	**<0.001**
	Earthworm species (E)	*4*	5	**0.001**
	E x L	*12*	3.6	**<0.001**
	Residuals	*75*		
**Bacterial abundance in litter**	Litter treatment (L)	*3*	99.57	**<0.001**
	Earthworm species (E)	*4*	8.87	**<0.001**
	E x L	*12*	8.7	**<0.001**
	Residuals	*75*		

Table S6 Estimated marginal means based on linear models for litter treatments (wheat straw, horse manure, legume leaves and rape leaves) and earthworm species (*Eisenia fetida*, *Lumbricus terrestris*, *Aporrectodea rosea*, *Aporrectodea caliginosa*, *Allolobophora chlorotica)*. Response variables include fungi-to-bacteria ratio (F/B), fungal abundance (F) and bacterial abundance (B) in litter or soil; df, degrees of freedom; CL, 95% confidence level estimated based on the "emmeans" package; P-values are derived from contrast against wheat straw nested within each earthworm species.

**Litter types**	**Means**	**Standard error**	**df**	**lower. CL**	**upper. CL**	**P-value (contrast vs. wheat straw)**	**Variables**	**Material**
Horse manure	-0.332	0.122	91	-0.576	-0.089	**0.008**	F/B	Litter
Legume leaves	0.465	0.127	91	0.213	0.717	**<0.001**	F/B	Litter
Rape leaves	0.490	0.121	91	0.249	0.731	**<0.001**	F/B	Litter
Horse manure	0.767	0.070	91	0.628	0.906	**<0.001**	F	Litter
Legume leaves	1.448	0.073	91	1.304	1.592	**<0.001**	F	Litter
Rape leaves	1.637	0.069	91	1.499	1.774	**<0.001**	F	Litter
Horse manure	1.099	0.102	91	0.897	1.301	**<0.001**	B	Litter
Legume leaves	0.983	0.105	91	0.774	1.192	**<0.001**	B	Litter
Rape leaves	1.147	0.101	91	0.947	1.346	**<0.001**	B	Litter
Horse manure	0.057	0.044	93	-0.030	0.144	0.195	F/B	Soil
Legume leaves	-0.026	0.044	93	-0.113	0.060	0.550	F/B	Soil
Rape leaves	0.040	0.044	93	-0.047	0.126	0.366	F/B	Soil
Horse manure	0.201	0.055	93	0.091	0.311	**<0.001**	F	Soil
Legume leaves	0.091	0.055	93	-0.019	0.201	0.106	F	Soil
Rape leaves	0.078	0.055	93	-0.033	0.188	0.165	F	Soil
Horse manure	0.144	0.039	93	0.067	0.222	**<0.001**	B	Soil
Legume leaves	0.117	0.039	93	0.039	0.195	**0.004**	B	Soil
Rape leaves	0.038	0.039	93	-0.040	0.116	0.335	B	Soil

Table. S7 Estimated marginal means based on linear models for litter treatments (wheat straw, horse manure, legume leaves and rape leaves) and earthworm species (*Eisenia fetida*, *Lumbricus terrestris*, *Aporrectodea rosea*, *Aporrectodea caliginosa*, *Allolobophora chlorotica).* Response variables include earthworm biomass (EM) and litter mass (LM); df, degrees of freedom; CL, 95% confidence level estimated based on the "emmeans" package; P-values derived from final against initial mass nested within each earthworm species.

Litter type	Earthworms	Means	Standard error	df	lower. CL	upper. CL	P-value (final vs. initial)	Variables
Legume leaves	*A. caliginosa*	0.347	0.036	472	0.277	0.417	**<0.001**	EM
Horse manure	*A. caliginosa*	0.185	0.036	472	0.115	0.255	**<0.001**	EM
Rape leaves	*A. caliginosa*	0.094	0.036	472	0.024	0.164	**0.009**	EM
Wheat straw	*A. caliginosa*	0.091	0.036	472	0.021	0.161	**0.011**	EM
Legume leaves	*A. chlorotica*	0.090	0.036	472	0.020	0.160	**0.012**	EM
Horse manure	*A. chlorotica*	0.041	0.036	472	-0.029	0.111	0.249	EM
Rape leaves	*A. chlorotica*	0.032	0.036	472	-0.039	0.102	0.377	EM
Wheat straw	*A. chlorotica*	0.018	0.036	472	-0.052	0.088	0.608	EM
Legume leaves	*A. rosea*	0.054	0.036	472	-0.016	0.124	0.128	EM
Horse manure	*A. rosea*	0.058	0.036	472	-0.012	0.128	0.102	EM
Rape leaves	*A. rosea*	0.043	0.036	472	-0.028	0.115	0.234	EM
Wheat straw	*A. rosea*	0.058	0.036	472	-0.013	0.130	0.110	EM
Legume leaves	*E. fetida*	0.112	0.037	472	0.039	0.185	**0.003**	EM
Horse manure	*E. fetida*	0.064	0.036	472	-0.006	0.134	0.074	EM
Rape leaves	*E. fetida*	0.134	0.037	472	0.061	0.207	**<0.001**	EM
Wheat straw	*E. fetida*	-0.050	0.037	472	-0.123	0.023	0.179	EM
Legume leaves	*L. terrestris*	0.756	0.036	472	0.686	0.826	**<0.001**	EM
Horse manure	*L. terrestris*	0.284	0.036	472	0.214	0.354	**<0.001**	EM
Rape leaves	*L. terrestris*	0.599	0.036	472	0.529	0.669	**<0.001**	EM
Wheat straw	*L. terrestris*	-0.012	0.036	472	-0.082	0.058	0.741	EM
Wheat straw	*E. fetida*	-0.660	0.132	80	-0.922	-0.397	**<0.001**	LM
Rape leaves	*E. fetida*	-7.100	0.132	80	-7.362	-6.837	**<0.001**	LM
Horse manure	*E. fetida*	-4.068	0.132	80	-4.331	-3.805	**<0.001**	LM
Legume leaves	*E. fetida*	-5.685	0.132	80	-5.948	-5.422	**<0.001**	LM
Wheat straw	*L. terrestris*	-1.000	0.132	80	-1.262	-0.737	**<0.001**	LM
Rape leaves	*L. terrestris*	-7.266	0.132	80	-7.529	-7.003	**<0.001**	LM
Horse manure	*L. terrestris*	-6.609	0.132	80	-6.872	-6.347	**<0.001**	LM
Legume leaves	*L. terrestris*	-7.446	0.132	80	-7.709	-7.183	**<0.001**	LM
Wheat straw	*A. rosea*	-0.768	0.132	80	-1.031	-0.505	**<0.001**	LM
Rape leaves	*A. rosea*	-4.738	0.132	80	-5.000	-4.475	**<0.001**	LM
Horse manure	*A. rosea*	-1.436	0.132	80	-1.699	-1.174	**<0.001**	LM

Legume leaves	*A. rosea*	-3.924	0.132	80	-4.186	-3.661	**<0.001**	LM
Wheat straw	*A. caliginosa*	-0.781	0.132	80	-1.044	-0.519	**<0.001**	LM
Rape leaves	*A. caliginosa*	-5.032	0.132	80	-5.295	-4.769	**<0.001**	LM
Horse manure	*A. caliginosa*	-3.879	0.132	80	-4.141	-3.616	**<0.001**	LM
Legume leaves	*A. caliginosa*	-5.544	0.132	80	-5.807	-5.281	**<0.001**	LM
Wheat straw	*A. chlorotica*	-0.795	0.132	80	-1.057	-0.532	**<0.001**	LM
Rape leaves	*A. chlorotica*	-4.941	0.132	80	-5.204	-4.678	**<0.001**	LM
Horse manure	*A. chlorotica*	-1.921	0.132	80	-2.184	-1.658	**<0.001**	LM
Legume leaves	*A. chlorotica*	-4.863	0.132	80	-5.126	-4.600	**<0.001**	LM

Table S8 Pairwise comparison based on linear models for litter treatments (wheat straw, horse manure, legume leaves and rape leaves) and earthworm species (*Eisenia fetida*, *Lumbricus terrestris*, *Aporrectodea rosea*, *Aporrectodea caliginosa*, *Allolobophora chlorotica).* Response variables include fungal abundance (F) and bacterial abundance (B) in litter or soil. Means represent estimated marginal means; df, degrees of freedom; P-values are derived from pairwise comparison between earthworm species nested within each litter treatment.

Contrast	Litter type	Means	Standard error	df	P-value	Variables	Material
A. caliginosa - A. chlorotica	Horse manure	0.890	0.401	77	0.184	F	Soil
A. caliginosa - A. rosea	Horse manure	1.126	0.378	77	0.031	F	Soil
A. caliginosa - E. fetida	Horse manure	0.050	0.378	77	1.000	F	Soil
A. caliginosa - L. terrestris	Horse manure	-0.736	0.378	77	0.303	F	Soil
A. chlorotica - A. rosea	Horse manure	0.236	0.401	77	0.977	F	Soil
A. chlorotica - E. fetida	Horse manure	-0.841	0.401	77	0.233	F	Soil
A. chlorotica - L. terrestris	Horse manure	-1.626	0.401	77	**0.001**	F	Soil
A. rosea - E. fetida	Horse manure	-1.077	0.378	77	**0.044**	F	Soil
A. rosea - L. terrestris	Horse manure	-1.862	0.378	77	**<0.001**	F	Soil
E. fetida - L. terrestris	Horse manure	-0.785	0.378	77	0.241	F	Soil
A. caliginosa - A. chlorotica	Legume leaves	0.142	0.378	77	0.996	F	Soil
A. caliginosa - A. rosea	Legume leaves	0.175	0.378	77	0.990	F	Soil
A. caliginosa - E. fetida	Legume leaves	-0.808	0.378	77	0.216	F	Soil
A. caliginosa - L. terrestris	Legume leaves	0.092	0.401	77	0.999	F	Soil
A. chlorotica - A. rosea	Legume leaves	0.034	0.378	77	1.000	F	Soil
A. chlorotica - E. fetida	Legume leaves	-0.949	0.378	77	0.099	F	Soil
A. chlorotica - L. terrestris	Legume leaves	-0.049	0.401	77	1.000	F	Soil
A. rosea - E. fetida	Legume leaves	-0.983	0.378	77	0.081	F	Soil
A. rosea - L. terrestris	Legume leaves	-0.083	0.401	77	1.000	F	Soil
E. fetida - L. terrestris	Legume leaves	0.900	0.401	77	0.175	F	Soil
A. caliginosa - A. chlorotica	Rape leaves	-0.082	0.378	77	1.000	F	Soil
A. caliginosa - A. rosea	Rape leaves	-0.140	0.378	77	0.996	F	Soil
A. caliginosa - E. fetida	Rape leaves	-0.595	0.401	77	0.577	F	Soil
A. caliginosa - L. terrestris	Rape leaves	-0.105	0.378	77	0.999	F	Soil
A. chlorotica - A. rosea	Rape leaves	-0.058	0.378	77	1.000	F	Soil
A. chlorotica - E. fetida	Rape leaves	-0.513	0.401	77	0.705	F	Soil
A. chlorotica - L. terrestris	Rape leaves	-0.023	0.378	77	1.000	F	Soil
A. rosea - E. fetida	Rape leaves	-0.455	0.401	77	0.788	F	Soil
A. rosea - L. terrestris	Rape leaves	0.035	0.378	77	1.000	F	Soil
E. fetida - L. terrestris	Rape leaves	0.491	0.401	77	0.739	F	Soil
A. caliginosa - A. chlorotica	Wheat straw	0.384	0.378	77	0.848	F	Soil

A. caliginosa - A. rosea	Wheat straw	-0.564	0.378	77	0.571	F	Soil
A. caliginosa - E. fetida	Wheat straw	0.097	0.378	77	0.999	F	Soil
A. caliginosa - L. terrestris	Wheat straw	-0.345	0.378	77	0.891	F	Soil
A. chlorotica - A. rosea	Wheat straw	-0.948	0.378	77	0.100	F	Soil
A. chlorotica - E. fetida	Wheat straw	-0.287	0.378	77	0.941	F	Soil
A. chlorotica - L. terrestris	Wheat straw	-0.729	0.378	77	0.312	F	Soil
A. rosea - E. fetida	Wheat straw	0.661	0.378	77	0.412	F	Soil
A. rosea - L. terrestris	Wheat straw	0.219	0.378	77	0.978	F	Soil
E. fetida - L. terrestris	Wheat straw	-0.442	0.378	77	0.769	F	Soil
A. caliginosa - A. chlorotica	Horse manure	-1.286	2.122	77	0.974	B	Soil
A. caliginosa - A. rosea	Horse manure	5.661	2.001	77	**0.046**	B	Soil
A. caliginosa - E. fetida	Horse manure	-8.134	2.001	77	**0.001**	B	Soil
A. caliginosa - L. terrestris	Horse manure	-6.982	2.001	77	**0.007**	B	Soil
A. chlorotica - A. rosea	Horse manure	6.947	2.122	77	**0.013**	B	Soil
A. chlorotica - E. fetida	Horse manure	-6.848	2.122	77	**0.015**	B	Soil
A. chlorotica - L. terrestris	Horse manure	-5.696	2.122	77	0.066	B	Soil
A. rosea - E. fetida	Horse manure	-13.795	2.001	77	**<0.001**	B	Soil
A. rosea - L. terrestris	Horse manure	-12.643	2.001	77	**<0.001**	B	Soil
E. fetida - L. terrestris	Horse manure	1.152	2.001	77	0.978	B	Soil
A. caliginosa - A. chlorotica	Legume leaves	-1.162	2.001	77	0.978	B	Soil
A. caliginosa - A. rosea	Legume leaves	1.189	2.001	77	0.976	B	Soil
A. caliginosa - E. fetida	Legume leaves	-13.170	2.001	77	**<0.001**	B	Soil
A. caliginosa - L. terrestris	Legume leaves	-8.084	2.122	77	**0.003**	B	Soil
A. chlorotica - A. rosea	Legume leaves	2.351	2.001	77	0.765	B	Soil
A. chlorotica - E. fetida	Legume leaves	-12.008	2.001	77	**<0.001**	B	Soil
A. chlorotica - L. terrestris	Legume leaves	-6.922	2.122	77	**0.014**	B	Soil
A. rosea - E. fetida	Legume leaves	-14.359	2.001	77	**<0.001**	B	Soil
A. rosea - L. terrestris	Legume leaves	-9.273	2.122	77	**<0.001**	B	Soil
E. fetida - L. terrestris	Legume leaves	5.086	2.122	77	0.127	B	Soil
A. caliginosa - A. chlorotica	Rape leaves	-3.038	2.001	77	0.554	B	Soil
A. caliginosa - A. rosea	Rape leaves	-1.792	2.001	77	0.898	B	Soil
A. caliginosa - E. fetida	Rape leaves	-5.478	2.122	77	0.084	B	Soil
A. caliginosa - L. terrestris	Rape leaves	-6.286	2.001	77	**0.020**	B	Soil
A. chlorotica - A. rosea	Rape leaves	1.246	2.001	77	0.971	B	Soil
A. chlorotica - E. fetida	Rape leaves	-2.440	2.122	77	0.780	B	Soil
A. chlorotica - L. terrestris	Rape leaves	-3.248	2.001	77	0.487	B	Soil
A. rosea - E. fetida	Rape leaves	-3.686	2.122	77	0.418	B	Soil
A. rosea - L. terrestris	Rape leaves	-4.494	2.001	77	0.174	B	Soil
E. fetida - L. terrestris	Rape leaves	-0.808	2.122	77	0.995	B	Soil
A. caliginosa - A. chlorotica	Wheat straw	1.244	2.001	77	0.971	B	Soil
A. caliginosa - A. rosea	Wheat straw	2.712	2.001	77	0.658	B	Soil
A. caliginosa - E. fetida	Wheat straw	-0.152	2.001	77	1.000	B	Soil
A. caliginosa - L. terrestris	Wheat straw	-2.852	2.001	77	0.613	B	Soil

A. chlorotica - A. rosea	Wheat straw	1.468	2.001	77	0.948	B	Soil
A. chlorotica - E. fetida	Wheat straw	-1.396	2.001	77	0.956	B	Soil
A. chlorotica - L. terrestris	Wheat straw	-4.096	2.001	77	0.254	B	Soil
A. rosea - E. fetida	Wheat straw	-2.864	2.001	77	0.610	B	Soil
A. rosea - L. terrestris	Wheat straw	-5.564	2.001	77	0.052	B	Soil
E. fetida - L. terrestris	Wheat straw	-2.700	2.001	77	0.661	B	Soil
A. caliginosa - A. chlorotica	Horse manure	-183.966	189.835	75	0.868	F	Litter
A. caliginosa - A. rosea	Horse manure	-60.869	178.978	75	0.997	F	Litter
A. caliginosa - E. fetida	Horse manure	46.713	178.978	75	0.999	F	Litter
A. caliginosa - L. terrestris	Horse manure	-192.094	178.978	75	0.820	F	Litter
A. chlorotica - A. rosea	Horse manure	123.097	189.835	75	0.966	F	Litter
A. chlorotica - E. fetida	Horse manure	230.678	189.835	75	0.743	F	Litter
A. chlorotica - L. terrestris	Horse manure	-8.129	189.835	75	1.000	F	Litter
A. rosea - E. fetida	Horse manure	107.582	178.978	75	0.975	F	Litter
A. rosea - L. terrestris	Horse manure	-131.225	178.978	75	0.948	F	Litter
E. fetida - L. terrestris	Horse manure	-238.807	178.978	75	0.671	F	Litter
A. caliginosa - A. chlorotica	Legume leaves	73.671	200.103	75	0.996	F	Litter
A. caliginosa - A. rosea	Legume leaves	-188.230	216.136	75	0.907	F	Litter
A. caliginosa - E. fetida	Legume leaves	-271.908	189.835	75	0.609	F	Litter
A. caliginosa - L. terrestris	Legume leaves	-129.880	189.835	75	0.959	F	Litter
A. chlorotica - A. rosea	Legume leaves	-261.902	216.136	75	0.745	F	Litter
A. chlorotica - E. fetida	Legume leaves	-345.579	189.835	75	0.370	F	Litter
A. chlorotica - L. terrestris	Legume leaves	-203.551	189.835	75	0.820	F	Litter
A. rosea - E. fetida	Legume leaves	-83.677	206.666	75	0.994	F	Litter
A. rosea - L. terrestris	Legume leaves	58.350	206.666	75	0.999	F	Litter
E. fetida - L. terrestris	Legume leaves	142.027	178.978	75	0.932	F	Litter
A. caliginosa - A. chlorotica	Rape leaves	450.172	178.978	75	0.098	F	Litter
A. caliginosa - A. rosea	Rape leaves	189.461	178.978	75	0.827	F	Litter
A. caliginosa - E. fetida	Rape leaves	-717.949	178.978	75	**0.001**	F	Litter
A. caliginosa - L. terrestris	Rape leaves	-450.097	178.978	75	0.098	F	Litter
A. chlorotica - A. rosea	Rape leaves	-260.710	178.978	75	0.594	F	Litter
A. chlorotica - E. fetida	Rape leaves	-1168.121	178.978	75	**<0.001**	F	Litter
A. chlorotica - L. terrestris	Rape leaves	-900.269	178.978	75	**<0.001**	F	Litter
A. rosea - E. fetida	Rape leaves	-907.410	178.978	75	**<0.001**	F	Litter
A. rosea - L. terrestris	Rape leaves	-639.558	178.978	75	**0.006**	F	Litter
E. fetida - L. terrestris	Rape leaves	267.852	178.978	75	0.568	F	Litter
A. caliginosa - A. chlorotica	Wheat straw	5.014	178.978	75	1.000	F	Litter
A. caliginosa - A. rosea	Wheat straw	-20.439	178.978	75	1.000	F	Litter
A. caliginosa - E. fetida	Wheat straw	-9.491	178.978	75	1.000	F	Litter
A. caliginosa - L. terrestris	Wheat straw	-34.676	178.978	75	1.000	F	Litter
A. chlorotica - A. rosea	Wheat straw	-25.453	178.978	75	1.000	F	Litter
A. chlorotica - E. fetida	Wheat straw	-14.505	178.978	75	1.000	F	Litter
A. chlorotica - L. terrestris	Wheat straw	-39.690	178.978	75	1.000	F	Litter

A. rosea - E. fetida	Wheat straw	10.948	178.978	75	1.000	F	Litter
A. rosea - L. terrestris	Wheat straw	-14.237	178.978	75	1.000	F	Litter
E. fetida - L. terrestris	Wheat straw	-25.185	178.978	75	1.000	F	Litter
A. caliginosa - A. chlorotica	Horse manure	45.026	73.169	75	0.972	B	Litter
A. caliginosa - A. rosea	Horse manure	93.420	68.985	75	0.659	B	Litter
A. caliginosa - E. fetida	Horse manure	40.491	68.985	75	0.977	B	Litter
A. caliginosa - L. terrestris	Horse manure	29.755	68.985	75	0.993	B	Litter
A. chlorotica - A. rosea	Horse manure	48.393	73.169	75	0.964	B	Litter
A. chlorotica - E. fetida	Horse manure	-4.535	73.169	75	1.000	B	Litter
A. chlorotica - L. terrestris	Horse manure	-15.271	73.169	75	1.000	B	Litter
A. rosea - E. fetida	Horse manure	-52.928	68.985	75	0.939	B	Litter
A. rosea - L. terrestris	Horse manure	-63.665	68.985	75	0.887	B	Litter
E. fetida - L. terrestris	Horse manure	-10.737	68.985	75	1.000	B	Litter
A. caliginosa - A. chlorotica	Legume leaves	-173.596	77.127	75	0.173	B	Litter
A. caliginosa - A. rosea	Legume leaves	-132.636	83.307	75	0.507	B	Litter
A. caliginosa - E. fetida	Legume leaves	-16.079	73.169	75	1.000	B	Litter
A. caliginosa - L. terrestris	Legume leaves	542.079	73.169	75	**<0.001**	B	Litter
A. chlorotica - A. rosea	Legume leaves	40.960	83.307	75	0.988	B	Litter
A. chlorotica - E. fetida	Legume leaves	157.517	73.169	75	0.209	B	Litter
A. chlorotica - L. terrestris	Legume leaves	715.675	73.169	75	**<0.001**	B	Litter
A. rosea - E. fetida	Legume leaves	116.557	79.657	75	0.589	B	Litter
A. rosea - L. terrestris	Legume leaves	674.715	79.657	75	**<0.001**	B	Litter
E. fetida - L. terrestris	Legume leaves	558.158	68.985	75	**<0.001**	B	Litter
A. caliginosa - A. chlorotica	Rape leaves	-113.295	68.985	75	0.476	B	Litter
A. caliginosa - A. rosea	Rape leaves	-156.202	68.985	75	0.168	B	Litter
A. caliginosa - E. fetida	Rape leaves	-37.698	68.985	75	0.982	B	Litter
A. caliginosa - L. terrestris	Rape leaves	3.125	68.985	75	1.000	B	Litter
A. chlorotica - A. rosea	Rape leaves	-42.907	68.985	75	0.971	B	Litter
A. chlorotica - E. fetida	Rape leaves	75.597	68.985	75	0.808	B	Litter
A. chlorotica - L. terrestris	Rape leaves	116.419	68.985	75	0.448	B	Litter
A. rosea - E. fetida	Rape leaves	118.504	68.985	75	0.429	B	Litter
A. rosea - L. terrestris	Rape leaves	159.326	68.985	75	0.153	B	Litter
E. fetida - L. terrestris	Rape leaves	40.823	68.985	75	0.976	B	Litter
A. caliginosa - A. chlorotica	Wheat straw	-15.311	68.985	75	0.999	B	Litter
A. caliginosa - A. rosea	Wheat straw	-26.198	68.985	75	0.996	B	Litter
A. caliginosa - E. fetida	Wheat straw	-18.498	68.985	75	0.999	B	Litter
A. caliginosa - L. terrestris	Wheat straw	-57.439	68.985	75	0.920	B	Litter
A. chlorotica - A. rosea	Wheat straw	-10.887	68.985	75	1.000	B	Litter
A. chlorotica - E. fetida	Wheat straw	-3.187	68.985	75	1.000	B	Litter
A. chlorotica - L. terrestris	Wheat straw	-42.128	68.985	75	0.973	B	Litter
A. rosea - E. fetida	Wheat straw	7.701	68.985	75	1.000	B	Litter
A. rosea - L. terrestris	Wheat straw	-31.241	68.985	75	0.991	B	Litter
E. fetida - L. terrestris	Wheat straw	-38.942	68.985	75	0.980	B	Litter

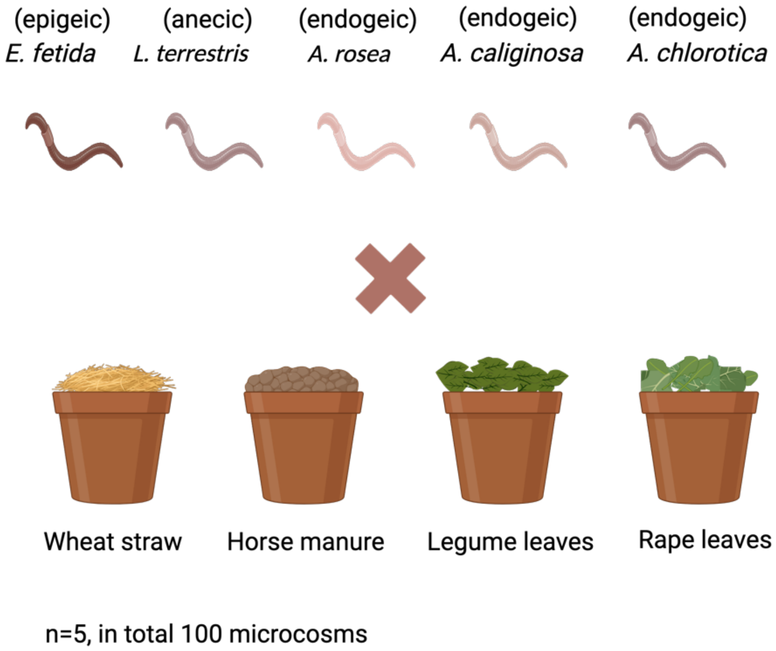

Figure S1. Schematic illustration of the full-factorial design with two treatments, i.e., litter types (four levels: wheat straw, horse manure, legume leaves, rape leaves) and earthworm species (five levels: *Eisenia fetida*, *Lumbricus terrestris*, *Aporrectodea rosea*, *Aporrectodea caliginosa*, *Allolobophora chlorotica).* The average C-to-N ratios of wheat straw, horse manure, legume leaves and rape leaves were 134.42 ± 0.77, 43.52 ± 0.69, 15.15 ± 0.03, and 12.28 ± 0.06, respectively. Based on the C-to-N ratios, we ranked the quality of litter types from low to high as follows: wheat straw < horse manure < legume leaves < rape leaves. The figure was created in BioRender (L. Zhong, 2025; https://BioRender.com/f01i953).

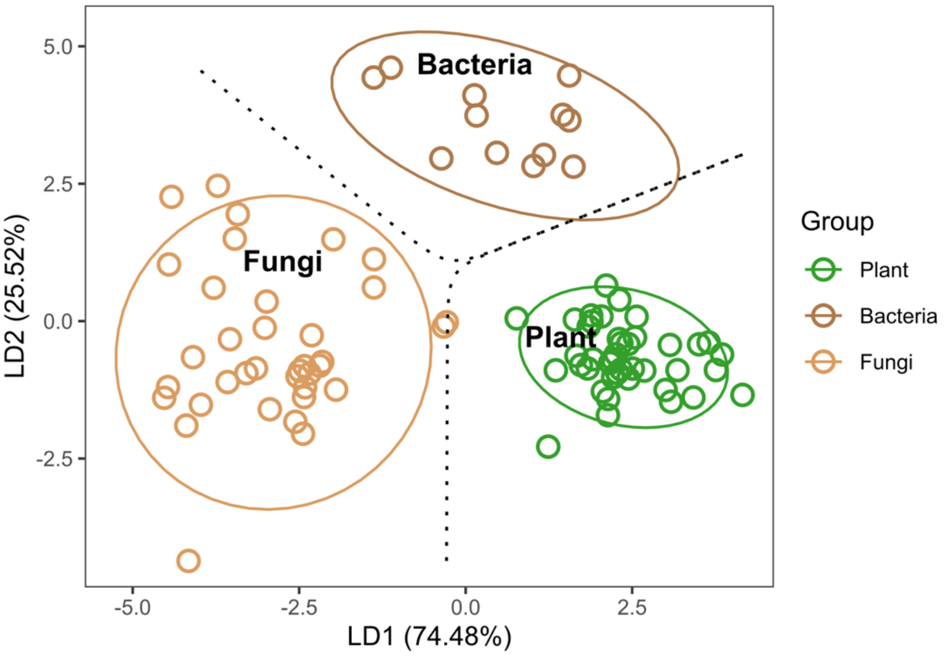

Figure S2. Aggregated basal resource profiles in linear discriminant space. Points represent $\delta^{13}C$ profiles of essential amino acids (leucine, isoleucine, threonine, phenylalanine and valine). Data are shown with 95% confidence ellipses for plants, bacteria and fungi.

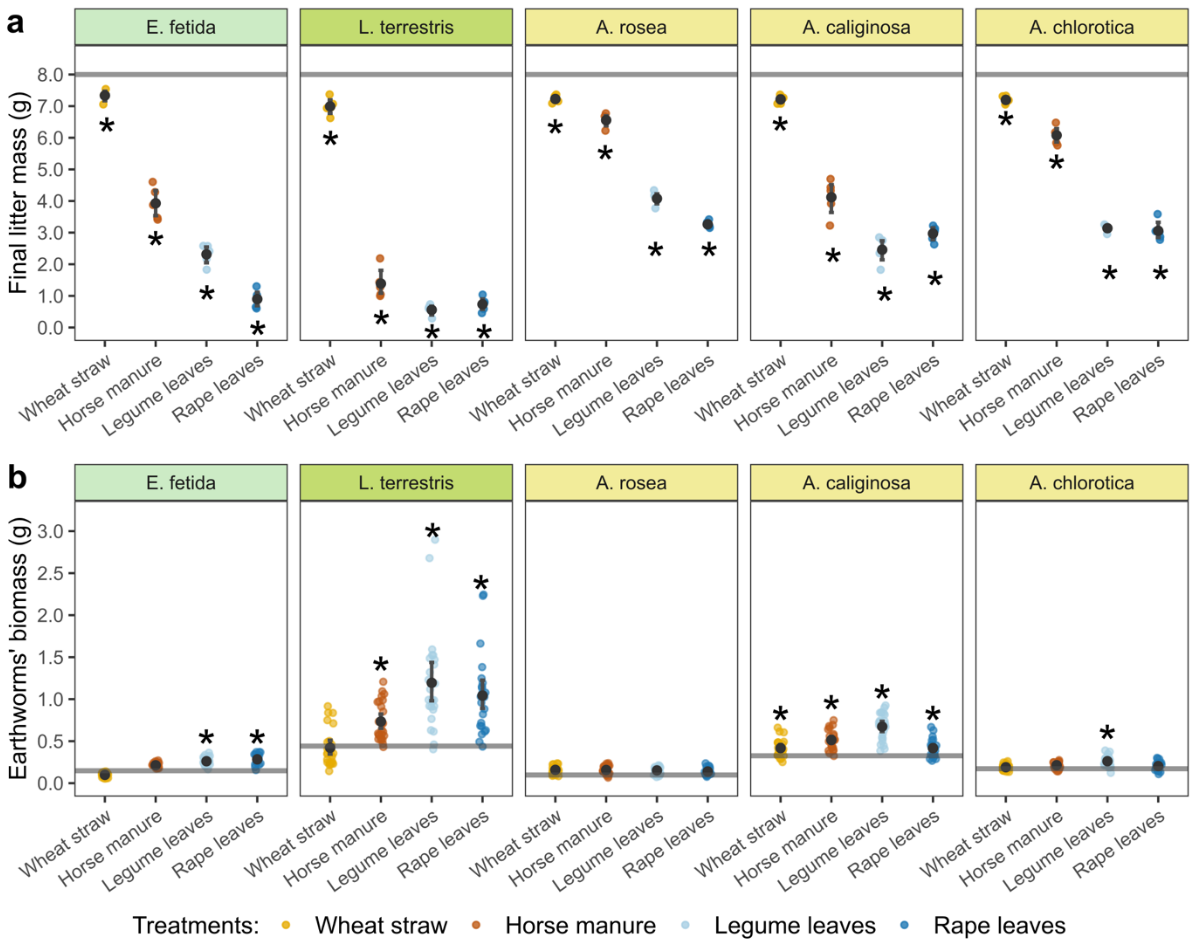

Figure S3. (a) Remaining litter mass of wheat straw, horse manure, legume leaves and rape leaves in treatments with different earthworm species (*Eisenia fetida*, *Lumbricus terrestris*, *Aporrectodea rosea*, *Aporrectodea caliginosa*, *Allolobophora chlorotica*). The grey line represents the mean initial litter mass. Asterisks indicate significant differences to the mean initial litter mass ($P < 0.05$; Table S7). **(b)** Biomass of earthworm species (*Eisenia fetida*, *Lumbricus terrestris*, *Aporrectodea rosea*, *Aporrectodea caliginosa*, *Allolobophora chlorotica*) in different litter treatments (wheat straw, horse manure, legume leaves, rape leaves). The grey line represents the arithmetic mean of the initial biomass of earthworms. Asterisks indicate significant differences to the initial biomass ($P < 0.05$; Table S7). Dots with respective colour indicate individual specimens, and the black dots the mean values, the error bar gives 95% confidence intervals.

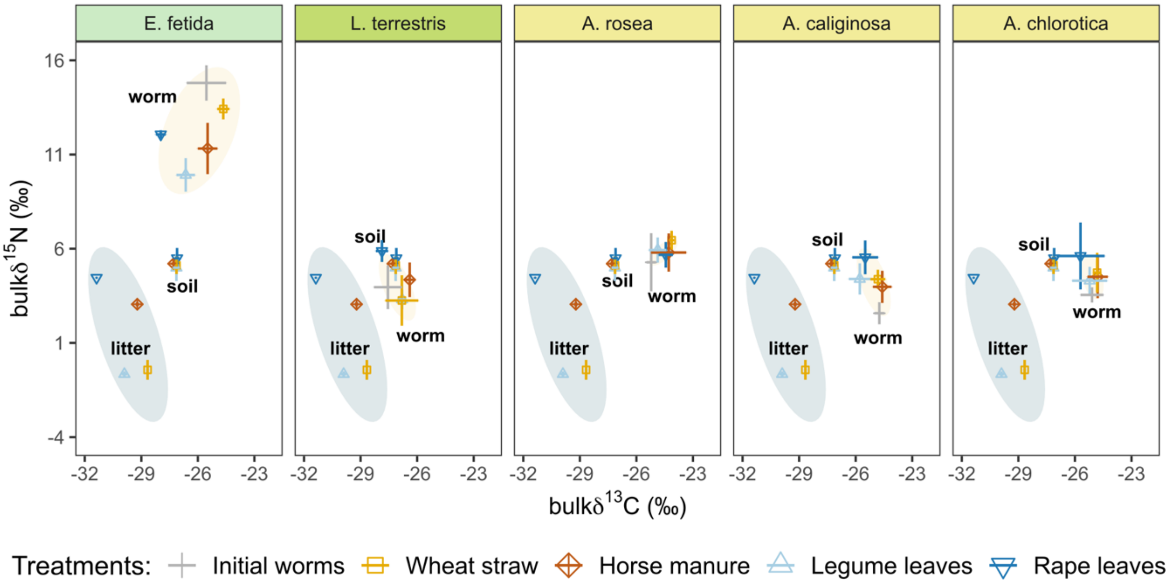

Figure S4. Bulk $\delta^{13}C$ and $\delta^{15}N$ values of earthworms (light yellow ellipse), litters (grey ellipse) and soil (light purple ellipse) in treatments with wheat straw (yellow; square), horse manure (brown; diamond), legume leaves (light blue; triangle) and rape leaves (dark blue; angle down) as well as earthworms prior to placement into the microcosms (initial worms, grey; cross). Symbols represent the mean and lines the 95% confidence intervals of five replicates. Significant differences are indicated by non-overlapping confidence intervals between two bars. Ellipses represent 75% confidence intervals.

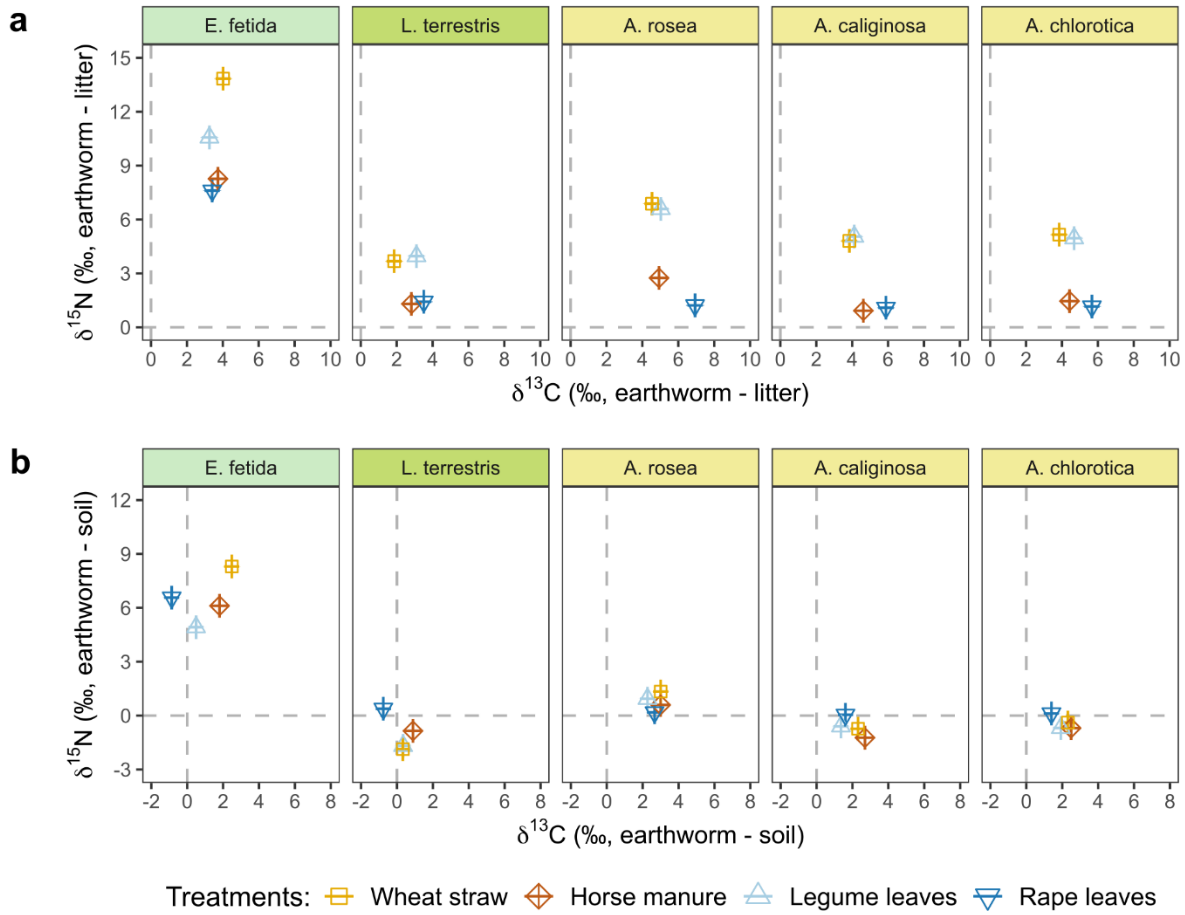

Figure S5. (**a**) Bulk $\delta^{13}C$ and $\delta^{15}N$ values of earthworm species (*Eisenia fetida*, *Lumbricus terrestris*, *Aporrectodea rosea*, *Aporrectodea caliginosa*, *Allolobophora chlorotica*) as calibrated by respective litter materials. (**b**) Bulk $\delta^{13}C$ and $\delta^{15}N$ values of the five earthworm species studied as calibrated by the respective soil. Colour and shape coding represent the litter treatments; wheat straw (yellow; square), horse manure (brown; diamond), legume leaves (light blue; triangle) and rape leaves (dark blue; angle down). Symbols represent means and lines 95% confidence ranges of five replicates. Significant differences are indicated by non-overlapping confidence intervals.

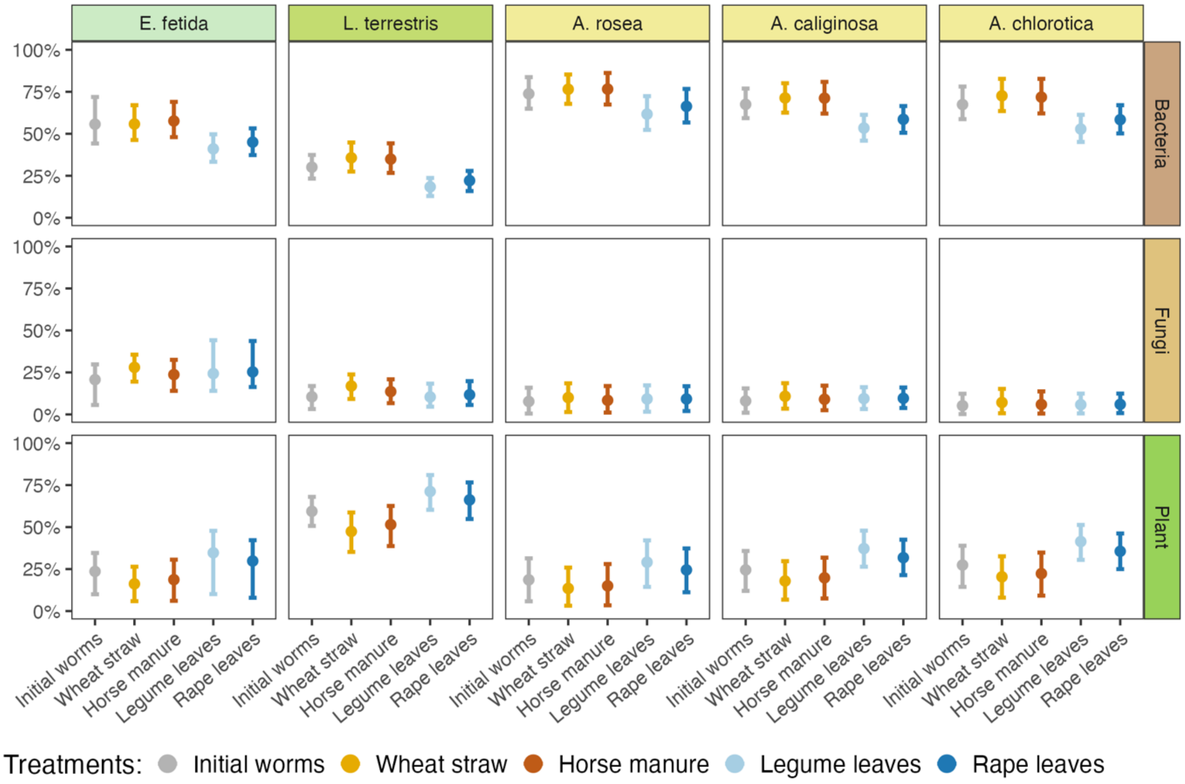

Figure S6. Mean (± 95% confidence interval) relative contribution of bacterial-, plant- and fungal-derived essential amino acids to the diet of earthworms in the treatments with wheat straw (yellow), horse manure (brown), legume leaves (light blue) and rape leaves (dark blue) as well as of earthworms before placement into the microcosms (initial; grey) as estimated by Bayesian mixing models based on mean-centred $\delta^{13}C$ values of the three most informative essential amino acids leucine, phenylalanine and valine. Significant differences are indicated by non-overlapping confidence intervals between bars.

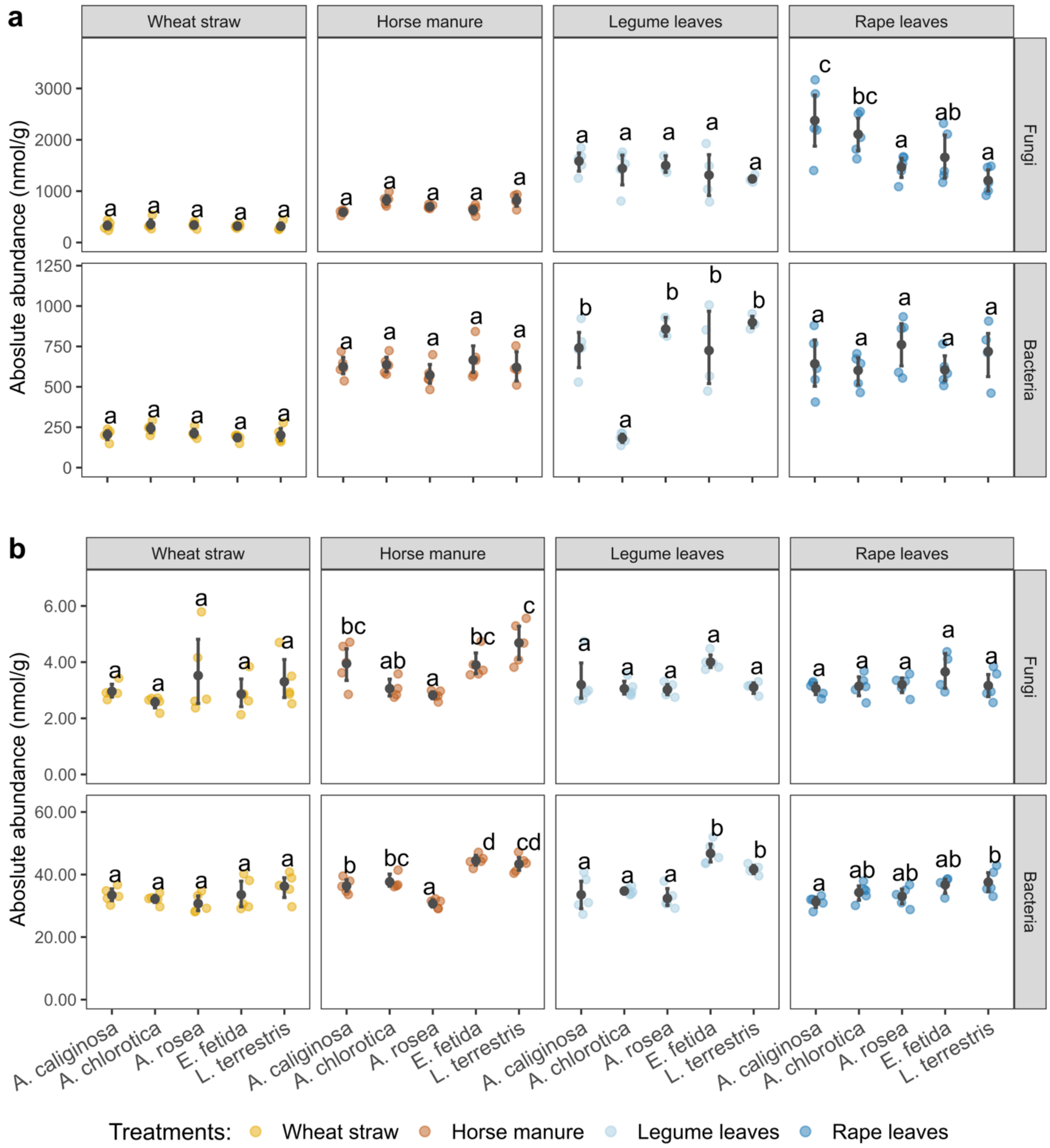

Figure S7. Absolute abundance of fungal and bacterial phospholipid fatty acid (PLFA) markers in litter **(a)** and soil **(b)** in litter (wheat straw, horse manure, legume leaves, rape leaves) and earthworm species (*Eisenia fetida*, *Lumbricus terrestris*, *Aporrectodea rosea*, *Aporrectodea caliginosa*, *Allolobophora chlorotica*) treatments. Dots with representative colour indicate

individual specimens and black dots the mean values; the error bar gives the 95% confidence intervals. Different letters within litter treatments indicate significant differences between earthworm species treatments ($P < 0.05$; Tukey's HSD test; Table S8).
