# Supplementary material for: High litter quality enhances plant energy channeling by soil macro‐detritivores and lowers their trophic position
Source: Ecology. 2025 Feb 23;106(2):e70004. doi: 10.1002/ecy.70004 (PMC11848239; doi:10.1002/ecy.70004)
Supplement: Supplementary file 2 — Appendix S2. [file ECY-106-e70004-s001.pdf]

Journal: Ecology

Manuscript type: Article

High litter quality enhances plant energy channelling by soil macro-detritivores and lowers their trophic position

Authors: Linlin Zhong*, Thomas Larsen, Jing-Zhong Lu, Stefan Scheu, Melanie M. Pollierer

*Corresponding author (E-mail: linlin.zhong@gwdg.de)

Appendix S2

Section S1: Bulk stable isotope analysis

For bulk stable isotope analysis, 100-200 μg dried earthworm tissue, 1.2-2.1 mg dried litter material and 9.0-10.5 mg dried soil were weighed into tin capsules. The natural abundance of bulk stable isotopes of both C and N of earthworms, litter and soil were measured by a coupled system of an elemental analyser (Flash 2000, Thermo Fisher Scientific, Cambridge, UK) and an isotope ratio mass spectrometer (Delta V Advantage, Thermo Electron, Bremen, Germany). Vienna Pee Dee Belemnite and atmospheric nitrogen were used as standards, respectively. Acetanilide (C_8H_9NO, Merck, Darmstadt, Germany) was used for internal calibration.

Section S2: Amino acids extraction

2-4 mg dried earthworm tissue and ca. 12 mg dried litter materials were weighed into Pyrex culture tubes and flushed with N_2 gas, sealed and hydrolysed in 1 ml 6 N HCl at 110°C in a heating block for 20 h. After hydrolysis, the lipophilic compounds of samples were removed by adding 2 ml hexane/DCM to the Pyrex tubes, which were then flushed shortly with N_2 gas and sealed prior to vortexing for 30 s. The aqueous phase was then filtered through a Pasteur pipette lined with glass wool that had been heated at 450°C to remove potential contaminations. Samples were transferred into 4 ml dram vials before evaporation to dryness under N_2 gas at 110°C in a heating block for 30 min. Nor-leucine was added to each sample as an internal standard. Then dried samples were methylated with acidified methanol and acetylated with a mixture of acetic anhydride, triethylamine and acetone (1:2:5) to produce N-acetyl methyl ester derivatives. Samples were flushed with N_2 gas and sealed before methylation and acetylation to

reduce oxidation during derivatization. Pure gas with known $\delta^{13}C$ and $\delta^{15}N$ values was derivatised and analysed along with the samples to account for added carbon and isotope fractionation during the analysis.

Section S3: Compound-specific isotope analysis of amino acids

Amino acid derivatives were injected into a Thermo Finnigan Trace 1310 gas chromatograph (GC) coupled via a GP interface to a Delta Plus mass spectrometer (IRMS, Finnigan, Bremen, Germany). The gas chromatograph was equipped with an Agilent J&W VF-35ms GC column (30 m × 0.32 mm × 1.00 μm). The N isotope composition of amino acids was expressed relative to atmospheric nitrogen by normalizing measured values (vs. reference gas) using scales derived from known $\delta^{15}N$ values of the reference mixture, i.e., a mix of standard amino acids with known $\delta^{15}N$ value including alanine (*Ala*), asparagine/aspartic acid (*Asp*), glutamine/glutamic acid (*Glu*), glycine (*Gly*), histidine (*His*), isoleucine (*Ile*), leucine (*Leu*), lysine (*Lys*), methionine (*Met*), phenylalanine (*Phe*), threonine (*Thr*), tyrosine (*Tyr*) and valine (*Val*). Carbon isotope composition was corrected for carbon added during derivatization following O'Brien et al. (2002) and was expressed relative to Vienna Pee Dee Belemnite.

Section S4: Phospholipid fatty acids analysis

2 g fresh soil and 0.18-0.26 g dried litter materials were extracted by Bligh/Dyer solvent [chloroform, methanol, citrate buffer (pH 4); 1:2:0.8], chloroform and distilled water. Samples were then dried in a vacuum rotator (RVC 2-25, CHRIST®, Buddeberg, Mannheim, Germany). Then, chloroform was added to each sample which was then fractionated into neutral lipids, glycolipids and phospholipids by successive elution through silica acid columns (0.5 g silicic

acid, 3 ml; HF BOND ELUT-SI, Varian Inc., Darmstadt, Germany) with methanol, chloroform and distilled water. Phospholipids were then subjected to mild alkaline methanolysis and dissolved in 100 µl isooctane.

Fatty acid methyl esters were identified by chromatographic retention time according to standards (FAME CRM47885, C11 to C24; BAME 47080-U, C11 to C20; Sigma-Aldrich, Darmstadt, Germany) via gas chromatography using a GC- FID Clarus 500 (PerkinElmer Corporation, Norwalk, USA) equipped with a HP-5 capillary column (30 m x 0.32 mm i.d., film thickness 0.25 mm). Samples were injected in splitless mode via a split/splitless inlet. The temperature started with 60°C (held for 1min) and increased by 30°C/min to 160°C followed by 3°C/min to 260°C. The injection temperature was 250°C and helium was used as carrier gas.

References

O'Brien, D. M., M. L. Fogel, and C. L. Boggs. 2002. "Renewable and nonrenewable resources: Amino acid turnover and allocation to reproduction in Lepidoptera". *Proceedings of the National Academy of Sciences of the United States of America* 99:4413–4418.
